# Targeting LOXL2 for cardiac interstitial fibrosis and heart failure treatment

**DOI:** 10.1038/ncomms13710

**Published:** 2016-12-14

**Authors:** Jin Yang, Konstantinos Savvatis, Jong Seok Kang, Peidong Fan, Hongyan Zhong, Karen Schwartz, Vivian Barry, Amanda Mikels-Vigdal, Serge Karpinski, Dmytro Kornyeyev, Joanne Adamkewicz, Xuhui Feng, Qiong Zhou, Ching Shang, Praveen Kumar, Dillon Phan, Mario Kasner, Begoña López, Javier Diez, Keith C. Wright, Roxanne L. Kovacs, Peng-Sheng Chen, Thomas Quertermous, Victoria Smith, Lina Yao, Carsten Tschöpe, Ching-Pin Chang

**Affiliations:** 1 Krannert Institute of Cardiology and Division of Cardiology, Department of Medicine, Indiana University School of Medicine, Indianapolis, Indiana 46202, USA; 2 Department of Biochemistry and Molecular Biology, Indiana University School of Medicine, Indianapolis, Indiana 46202, USA; 3 Department of Medical and Molecular Genetics, Indiana University School of Medicine, Indianapolis, Indiana 46202, USA; 4 Department of Cardiology, Campus Virchow-Klinikum, Charité University Medicine Berlin, 13353 Berlin, Germany; 5 Berlin-Brandenburg Centre for Regenerative Therapies, Charité University Medicine Berlin, 10117 Berlin, Germany; 6 Gilead Sciences Inc., Foster City, California 94404, USA; 7 Division of Cardiovascular Medicine, Stanford University, Stanford, California 94305, USA; 8 Program of Cardiovascular Diseases, Centre for Applied Medical Research, Department of Cardiology and Cardiac Surgery, University Clinic, University of Navarra, 31008 Pamplona, Spain; 9 DZHK, German Centre for Cardiovascular Research, Partner Site Berlin – Charité, 13347 Berlin, Germany

## Abstract

Interstitial fibrosis plays a key role in the development and progression of heart failure. Here, we show that an enzyme that crosslinks collagen—Lysyl oxidase-like 2 (Loxl2)—is essential for interstitial fibrosis and mechanical dysfunction of pathologically stressed hearts. In mice, cardiac stress activates fibroblasts to express and secrete Loxl2 into the interstitium, triggering fibrosis, systolic and diastolic dysfunction of stressed hearts. Antibody-mediated inhibition or genetic disruption of Loxl2 greatly reduces stress-induced cardiac fibrosis and chamber dilatation, improving systolic and diastolic functions. Loxl2 stimulates cardiac fibroblasts through PI3K/AKT to produce TGF-β2, promoting fibroblast-to-myofibroblast transformation; Loxl2 also acts downstream of TGF-β2 to stimulate myofibroblast migration. In diseased human hearts, LOXL2 is upregulated in cardiac interstitium; its levels correlate with collagen crosslinking and cardiac dysfunction. LOXL2 is also elevated in the serum of heart failure (HF) patients, correlating with other HF biomarkers, suggesting a conserved LOXL2-mediated mechanism of human HF.

## Abstract (web summary)

Lysyl oxidase-like 2 (LOXL2) is an enzyme that promotes scaffolding of extracellular matrix proteins. Here the authors show that LOXL2 is crucial for pressure-overload induced cardiac fibrosis, and that antibody-mediated inhibition or genetic disruption of *Loxl2* in mice shows therapeutic potential for treatment of cardiac fibrosis.

Heart failure (HF) is a leading cause of death, with a mortality rate of ∼50% within five years of diagnosis^1^. This high mortality rate reflects inadequacy of modern therapy and calls for a new mechanistic paradigm for treatment. A major cause of cardiac dysfunction is the adverse tissue remodelling with interstitial fibrosis^2^,^3^,^4^,^5^,^6^, caused by various pathological insults that include hypertension and myocardial infarction, with the extent of interstitial fibrosis prognosticating clinical outcomes of the diseased heart^6^. Current therapies that improve HF survival primarily target the pathogenic mechanisms that occur within cardiomyocytes but not those mechanisms that take place outside cardiomyocytes in the interstitial space that houses collagen and fibroblasts. This lack of direct therapy against interstitial mechanism of HF may contribute to the persistently high morbidity and mortality of HF.

The major causes of death in HF are ventricular arrhythmias and pump failure. Although the arrhythmic death can be effectively prevented by implantable cardiac defibrillators, death from pump failure has become an even more pressing issue. Cardiac systolic and diastolic pump functions are both compromised in patients suffering from HF with reduced ejection fraction (HFrEF). However, current therapies only have moderate efficacy in systolic dysfunction and are ineffective for diastolic pump failure. For patients having HF with preserved ejection fraction (HFpEF), who have primarily diastolic dysfunction, there is no proven therapy that can improve diastolic function and long-term outcome. Therefore, an urgent need remains for novel, complementary medical therapies, which target maladaptive left ventricular tissue remodelling/fibrosis to improve systolic and diastolic functions of failing hearts^2^.

Interstitial fibrosis plays a key role in the development and progression of HF by causing adverse mechanical and electrical disturbances in the diseased hearts^2^. Interstitial fibrosis impairs the electromechanical coordination between cardiomyocytes to compromise systolic contractility. Fibrosis also increases ventricular stiffness and impairs diastolic relaxation and filling.

We found that Lysyl oxidase-like 2 (LOXL2) is upregulated in the interstitium of diseased mouse and human hearts. Increased LOXL2 expression leads to increased TGF-β2 production, triggering the formation and migration of myofibroblasts with enhanced collagen deposition and crosslinking in the hypertrophic regions of stressed hearts. These effects result in interstitial fibrosis and cardiac dysfunction. The concept of targeting LOXL2 in HF is supported by our preclinical data from cell-based assays, pharmacological studies and mouse genetic models, as well as by the clinical evidence of elevated cardiac tissue and serum LOXL2 in HF patients and correlation of tissue LOXL2 level with fibrosis and cardiac physiological changes in HF patients. The efficacy of anti-LOXL2 antibody and genetic LOXL2 disruption in relieving cardiac interstitial fibrosis and diastolic abnormalities is particularly salient, given the lack of direct therapy against cardiac fibrosis, and the increasing emphasis on diastolic dysfunction as an integral part of the heart failure syndrome.

## Results

### Loxl2 proteins are elevated in the stressed mouse hearts

**Figure 1:**
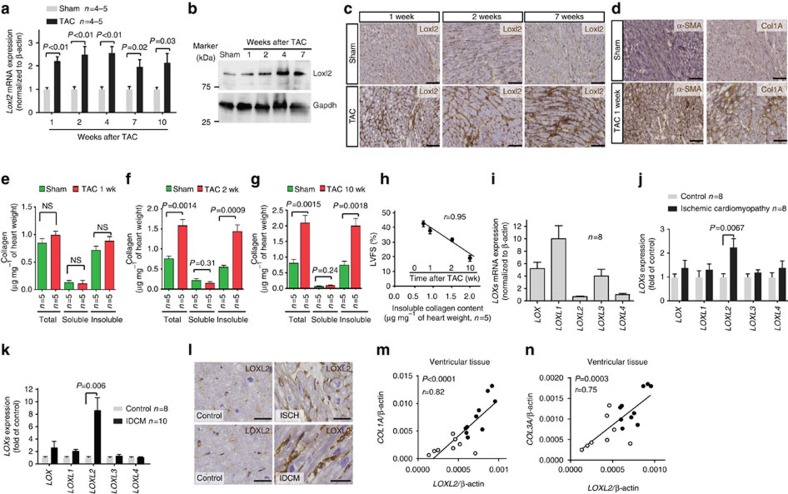
LOXL2 correlates with cardiac dysfunction in both mouse model and human patients. (**a**) Quantification of *Loxl2* mRNA expression in the heart 1–10 weeks after sham/TAC operation. *n*=4–5 mice per group. *P* value: Student's *t*-test. Error bar: standard error of the mean (s.e.m.). (**b**) Western blot analysis of Loxl2 protein in the mice heart ventricles 1–7 weeks after sham/TAC operation. *P* value: Student's *t*-test. Error bar: s.e.m. (**c**) Representative immunostaining of Loxl2 in the heart 1, 2 and 7 weeks after sham/TAC operation. Scale bars, 100 μm. Blue: haematoxylin. Brown: Loxl2. (**d**) Representative immunostaining of Col1A and α-SMA in the heart 1 week after sham/TAC operation. Scale bars, 100 μm. Blue: haematoxylin. Brown: Col1A/α-SMA. (**e**–**g**) Determination of total, soluble and insoluble collagen content of left ventricles from sham- or TAC-operated mice 1, 2 and 10 weeks after the procedure (*n*=5 in each group). The amounts of total and soluble collagen were determined by measuring hydroxyproline content of total and pepsin-acid solubilized left ventricles, respectively. The amount of insoluble collagen was calculated by subtracting the amount of soluble collagen from total collagen. (**h**) Correlation of left ventricular fractional shortening (LVFS) with the insoluble collagen content of left ventricles after sham or TAC operation (*n*=5 per group). *r*: Pearson coefficient. (**i**–**k**) mRNA quantification of *LOX* genes in ventricular tissues of healthy human donor hearts (*n*=8; **i**) or from patients with ischaemic (*n*=8; **j**) or idiopathic dilated (*n*=10; **k**) cardiomyopathy. IDCM, idiopathic dilated cardiomyopathy. *P* value: Student's *t*-test. Error bar: s.e.m. (**l**) Immunostaining of LOXL2 (brown) in human heart tissues obtained from control subjects and patients with ischaemic (ISCH) or idiopathic dilated cardiomyopathy (IDCM). Scale bars, 20 μm. Blue: haematoxylin. Brown: Loxl2. (**m**,**n**) Correlation of the mRNA level of *LOXL2* with *COL1A* (**m**) and *COL3A* (**n**) in the ventricular tissues of patients with idiopathic dilated cardiomyopathy (IDCM). *r*: Pearson coefficient. Black circles: cardiomyopathy tissues (*n*=10). Open circles: control heart tissues (*n*=8).

We used transaortic constriction (TAC) to cause pressure overload and hypertrophy of the hearts of male mice at 6–8 weeks of age^7^,^8^. Quantitative PCR (qPCR), western blot analysis and immunostaining of left ventricles showed that Loxl2 was minimally expressed in sham-operated hearts, but it was highly upregulated within the first week of TAC, and the Loxl2 upregulation persisted for >10 weeks after TAC (Fig. 1a–c, Supplementary Fig. 1a). Genes that encode other LOX isoforms (*Lox*, *Loxl1*, *Loxl3* and *Loxl4*) were also upregulated in the stressed hearts but to a lesser extent and consistency than *Loxl2* (Supplementary Fig. 1b–e). In TAC-stressed hearts, Loxl2 proteins were present in the interstitial space (Fig. 1c), which contains fibroblasts and collagen fibres. The upregulation of Loxl2 in the interstitium was accompanied by transdifferentiation of fibroblasts into myofibroblasts that are highly migratory (marked by expression of α-smooth muscle actin (α-SMA)) and synthetic of collagen (marked by expression of collagen, type I, alpha 1 (Col1A); Fig. 1d). The interstitium of TAC-stressed hearts, therefore, contains increased Loxl2 and collagen-producing myofibroblasts, providing the enzyme (Loxl2) and substrates (collagen) required for collagen crosslinking. Indeed, TAC-stressed hearts showed increased interstitial collagen, which was primarily insoluble (crosslinked; Fig. 1e–g), and such TAC-induced progressive increase of crosslinked collagen amount correlated strongly and linearly with the decline of left ventricular fractional shortening (LVFS) over time (Fig. 1h, *r*=0.95). Collectively, these findings suggest a role of Loxl2 in stress-induced interstitial collagen deposits and cardiac dysfunction.

### LOXL2 proteins are increased in the diseased human hearts

We next tested whether LOXL2 was upregulated in diseased human hearts. RT–qPCR of heart tissues showed that the expression of *LOXL2* and *LOXL4* was low in healthy human hearts, whereas the other isoforms *LOX*, *LOXL1* and *LOXL3* were more abundant (Fig. 1i). However, in human hearts with ischaemic or non-ischaemic dilated cardiomyopathy, only *LOXL2*, but not other LOX isoforms (*LOX*, *LOXL1*, *LOXL3* and *LOXL4*), was highly upregulated (Fig. 1j,k). Immunostaining verified the upregulation of LOXL2 protein in the interstitium of diseased human hearts (Fig. 1l). Quantification of messenger RNA (mRNA) revealed that *LOXL2* expression correlated well with the expression of fibrillar collagen genes (*COL1A* and *COL3A*) in ventricular tissues of healthy donor hearts (*n*=8) and of hearts from patients with idiopathic dilated cardiomyopathy (IDCM, *n*=10; Fig. 1m,n and Table 1). The clear separation of control and cardiomyopathy samples in the LOXL2–COLLAGEN correlation graphs indicates a functional role of LOXL2 elevation in the pathogenesis of human fibrotic hearts (Fig. 1m,n). Collectively, these findings show a specific elevation of the LOXL2 isoform in the interstitial space of diseased human hearts.

### LOXL2 is elevated in the serum of patients with heart failure

**Figure 2:**
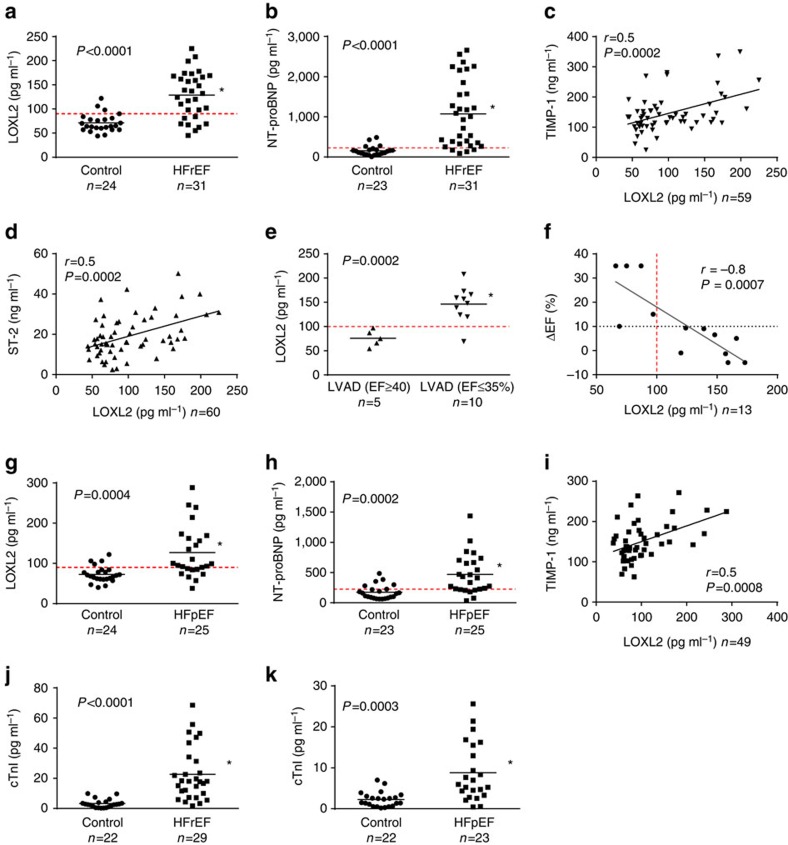
Serum LOXL2 as a biomarker for HFrEF and HFpEF. (**a**) Serum LOXL2 was measured by a customized ELISA-based assay. The red-dashed line represents a cutoff level of LOXL2 at 90 pg ml^−1^. The mean of each group is indicated by a horizontal line in the graph. *P<0.05 using unpaired Student's *t*-test. (**b**) Plasma NT-proBNP level was measured by Luminex. The red-dashed line represents a cutoff level of NT-proBNP at 225 pg ml^−1^. The mean of each group is indicated by a horizontal line in the graph. *P<0.05 using unpaired Student's *t*-test. (**c**) Correlation between serum LOXL2 and TIMP-1 measured by ELISA. The two-tailed *P* value for Pearson correlation was calculated using GraphPad Prism. (**d**) Correlation between serum LOXL2 and ST-2 measured by ELISA. The two-tailed *P* value for Pearson correlation was calculated using GraphPad Prism. (**e**) Serum LOXL2 versus post-LVAD EF ≤35% and ≥40%. LVAD: left ventricular assist device. The mean of each group is indicated by a horizontal line in the graph. *P<0.05 using unpaired Student's *t*-test. (**f**) Serum LOXL2 versus the degree of EF recovery of patients following LVAD therapy. The red-dashed line represents a cutoff level of LOXL2 at 100 pg ml^−1^. The two-tailed *P* value for Pearson correlation was calculated using GraphPad Prism. (**g**) Serum LOXL2 measured in HFpEF patients. The red-dashed line represents a cutoff level of LOXL2 at 90 pg ml^−1^. The mean of each group is indicated by a horizontal line in the graph. *P<0.05 using unpaired Student's *t*-test. (**h**) Plasma NT-proBNP level was measured by Luminex in HFpEF patients. The red-dashed line represents a cutoff level of NT-proBNP at 225 pg ml^−1^. The mean of each group is indicated by a horizontal line in the graph. *P<0.05 using unpaired Student's *t*-test. (**i**) Correlation between serum LOXL2 and TIMP-1 measured by ELISA. (**j**,**k**) Serum troponin I level in HFrEF (**j**) and HFpEF (**k**). The mean of each group is indicated by a horizontal line in the graph. *P<0.05 using unpaired Student's *t*-test.

To explore the potential use of LOXL2 as a HF biomarker, we tested whether LOXL2 proteins could be released from cardiac interstitium into the circulation. We first examined control subjects and patients with HFrEF (EF≤35%), the demographics of which was in Supplementary Table 1. We found that LOXL2 levels were elevated in the serum of HFrEF patients (129±9 pg ml^−1^, *n*=31) compared with control subjects (71±4 pg ml^−1^, *n*=24; Student's *t*-test, *P*<0.0001; Fig. 2a). A cutoff of LOXL2 at 90–100 pg ml^−1^ showed 88% specificity, 74% sensitivity and 80% accuracy in distinguishing HFrEF from the control subjects (Fig. 2a, red-dashed line). This high sensitivity/specificity was comparable to that of NT-proBNP, an established HF biomarker measured in the same serum samples (225 pg ml^−1^ cutoff measured by a research kit had 83% specificity, 87% sensitivity and 85% accuracy, Fig. 2b). These numbers of sensitivity and specificity were also comparable to the published results of NT-proBNP as a diagnostic HF biomarker.

Serum LOXL2 level correlated with that of TIMP-1, a tissue fibrosis marker^9^ (Pearson correlation, *r*=0.5, *P*<0.001; Fig. 2c) and ST-2, a cardiac remodelling and fibrosis marker (Pearson correlation, *r*=0.5, *P*<0.001; Fig. 2d). Of note, ST-2 plays a causal role in HF pathogenesis in animal models^10^, and ST-2 is a FDA-approved clinical serum HF biomarker that tracks the activity of cardiac fibroblasts and cardiomyocytes^11^,^12^. The correlations between LOXL2 and ST-2 and TIMP-1 suggest involvement of LOXL2 in the pathogenesis of human HFrEF.

Additional evidence implying a crucial role of LOXL2 in human HF came from our studies of patients with severe HFrEF receiving left ventricular assist device (LVAD; *n*=15; Supplementary Table 1). Those patients that had persistently low cardiac EF (≤35%), despite the LVAD therapy, exhibited elevated serum LOXL2 (146 pg ml^−1^), whereas those who showed significant EF recovery (≥40%) had serum LOXL2 comparable to control levels (76 pg ml^−1^) (Fig. 2e). Serum LOXL2 levels above 100 pg ml^−1^ had 90% sensitivity and 100% specificity of separating EF non-responders from responders following LVAD therapy (Fig. 2e). Moreover, EF recovery (ΔEF) before and after LVAD therapy correlated well with serum LOXL2 lowering (Pearson correlation, *r*=–0.8, *P*=0.001; Fig. 2f). A cutoff of LOXL2 above 100 pg ml^−1^ had 100% sensitivity and specificity in separating EF non-responder (ΔEF <10%) from responders (ΔEF ≥10%; Fig. 2f). These results not only suggest a potential use of LOXL2 as a biomarker in this clinical setting, but also implicate a causal role of LOXL2 in human HF.

Serum LOXL2 levels were also elevated in patients with heart failure with preserved ejection fraction (HFpEF; 127±13 pg ml^−1^, *n*=25) compared with the control subjects (73±4 pg ml^−1^, *n*=24; Student's *t*-test, *P*<0.0001; Fig. 2g). A cutoff of LOXL2 at 90 pg ml^−1^ displayed 83% specificity, 68% sensitivity and 76% accuracy in distinguishing HFpEF from control subjects (Fig. 2g, red-dashed line). This high sensitivity/specificity was again comparable to that of NT-proBNP, measured in the same serum samples (225 pg ml^−1^ cutoff had 74% specificity, 76% sensitivity and 75% accuracy, Fig. 2h). Moreover, significant correlation between sLOXL2 and tissue fibrotic marker TIMP-1 was observed in HFpEF (Fig. 2i). The serum samples of HFrEF and HFpEF patients were further validated by elevation of serum Troponin I levels measured by high-sensitivity troponin assays (Fig. 2j,k), suggesting continued cardiomyocyte injury in those patients. Given its accuracy in separating HFrEF or HFpEF patients from control subjects, serum LOXL2 may provide a new biomarker to track cardiac tissue remodelling and function in HF patients.

### LOXL2 correlates with diastolic dysfunction in HFpEF

**Figure 3:**
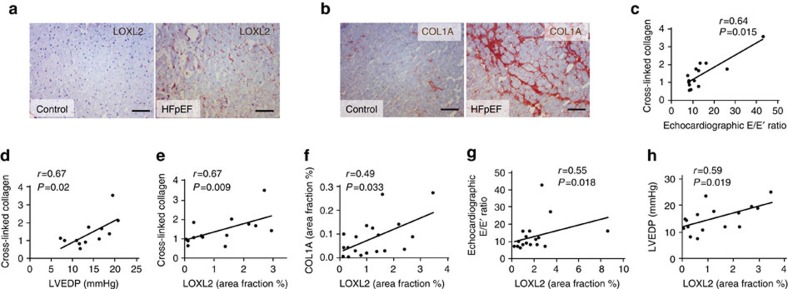
LOXL2 correlates with collagen crosslinking and diastolic abnormalities in HFpEF. (**a**,**b**) Immunostaining of LOXL2 (**a**) and COL1A (**b**) in heart tissues of control subjects and HfpEF patients. Scale bars, 100 μm. Blue: haematoxylin. Brown: LOXL2 (**a**) or COL1A (**b**). (**c**–**e**) Correlation of crosslinked collagen with echocardiographic E/E′ ratio (**c**), left ventricular end-diastolic pressure (LVEDP, **d**) and LOXL2 (**e**). *r*: Pearson coefficient. (**f**–**h**) Correlation of LOXL2 level with COL1A (**f**), echocardiographic E/E′ ratio (**g**) and left ventricular end-diastolic pressure (LVEDP, **h**) in human studies. *r*: Pearson coefficient.

To address the diastolic aspect of human heart failure, we studied control subjects (*n*=15) and HFpEF patients (*n*=24), whose demographic, echocardiographic and hemodynamic characteristics are listed in Table 2. The heart tissues from these patients showed intense interstitial fibrosis, coinciding with LOXL2 expression (Fig. 3a,b). In HFpEF patients, the extent of collagen crosslinking in the heart correlated well with diastolic relaxation abnormalities (measured by echocardiographic E/E′ ratio^13^) and with left ventricular end-diastolic pressure (LVEDP, measured by cardiac catheterization; Fig. 3c,d). These findings indicate a crucial role of crosslinked collagen in determining diastolic relaxation and filling pressure of the left ventricle. More importantly, cardiac LOXL2 levels correlated with the amount of crosslinked collagen, the severity of diastolic relaxation abnormalities (by E/E′ ratio), and the elevation of LVEDP (Fig. 3e–h). Because LOXL2 catalyses collagen crosslinking, these correlations support a LOXL2-mediated collagen mechanism that contributes to the diastolic dysfunction of HFpEF. Furthermore, the consistent findings of mouse and human heart tissue studies suggest an evolutionary conservation of LOXL2-based interstitial mechanism of cardiomyopathy.

### Loxl2 inhibition reduces stress-induced cardiac dysfunction

**Figure 4:**
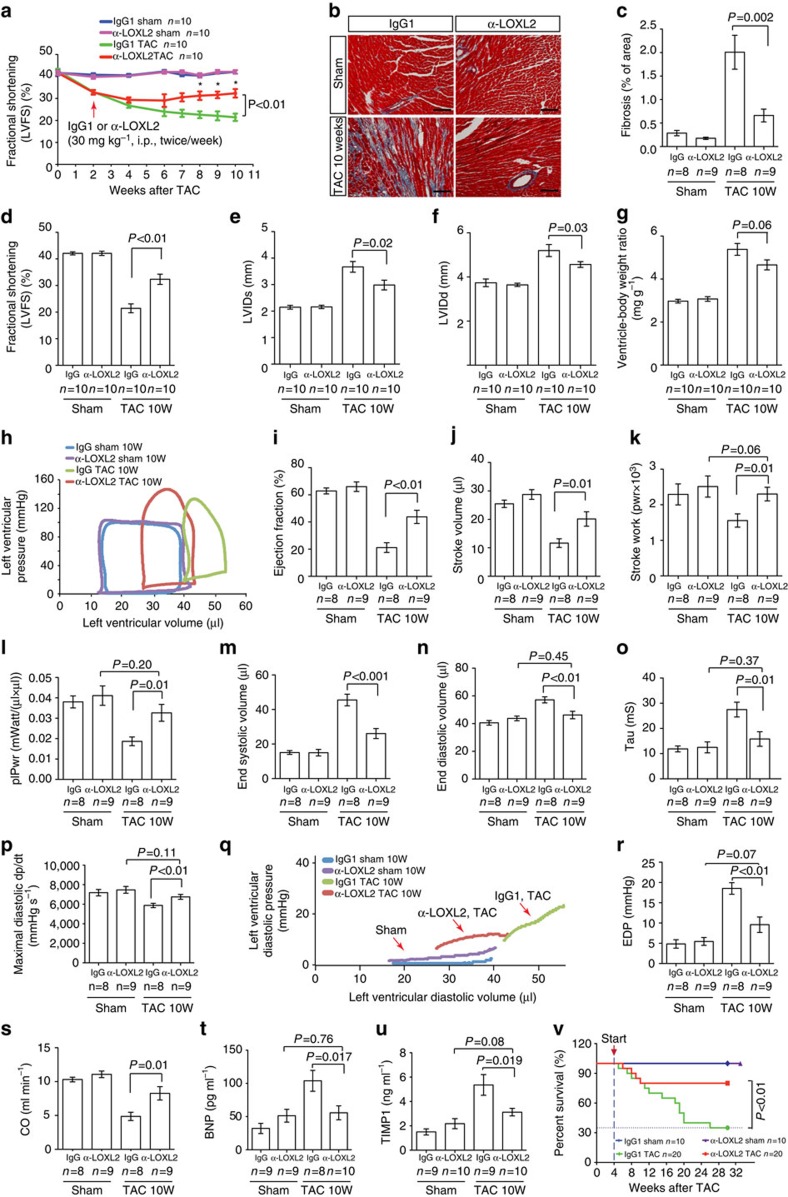
LOXL2 inhibition reduces interstitial fibrosis and reverses heart abnormalities. (**a**) Time course of left ventricular fractional shortening changes after sham/TAC operation with IgG1 or α-LOXL2 antibody treatment. i.p.: intraperitoneal injection. *P* value: Student's *t*-test. Error bar: s.e.m. (**b**,**c**) Trichrome staining (**b**) and quantification (**c**) of cardiac interstitial fibrosis in IgG1 or α-LOXL2-treated mice 10 weeks (10W) after sham or TAC operation. Scale bars, 100 μm. Red: cardiomyocytes. Blue: fibrosis. (**d**–**f**) Echocardiographic measurement of left ventricular fractional shortening (**d**), left ventricular internal diameter at systole (LVIDs, **e**) and at diastole (LVIDd, **f**) after 10 weeks of TAC. *P*-value: Student's *t*-test. Error bar: s.e.m. (**g**) Ventricle–body weight ratio of hearts harvested 10 weeks after sham or TAC operation. *P* value: Student's *t*-test. Error bar: s.e.m. (**h**) Representative cardiac pressure–volume relationships of IgG1- or α-LOXL2-treated mice 10 weeks after sham or TAC operation. (**i**–**p**) Quantification of ejection fraction (**i**), stroke volume (**j**), stroke work (**k**) and preload-adjusted maximal power (plPwr, **l**), end-systolic volume (**m**), end-diastolic volume (**n**), isovolumic relaxation time constant Tau (**o**) and maximal diastolic dp/dt (**p**) of IgG1- or α-LOXL2-treated mice 10 weeks after sham or TAC operation. *P* value: Student's *t*-test. Error bar: s.e.m. (**q**) Diastolic pressure–volume relationships of IgG1- or α-LOXL2-treated mice 10 weeks after sham or TAC operation. (**r**–**u**) End-diastolic pressure (EDP, **r**), cardiac output (**s**), serum brain natriuretic peptide (BNP, **t**) and serum tissue inhibitor of metalloproteinase 1 (TIMP1, **u**) of IgG1- or α-LOXL2-treated mice 10 weeks after sham or TAC operation. *P* value: Student's *t*-test. Error bar: s.e.m. (**v**) LOXL2 antibody significantly reduces mortality in TAC-operated mice. Data were analysed with Kaplan–Meier tests.

To determine a causative role of Loxl2 in interstitial fibrosis and heart failure, we used a LOXL2-specific neutralizing monoclonal antibody (AB0023, α-LOXL2; refs 6, 14) to inhibit Loxl2 activity in TAC-stressed mouse hearts. TAC and sham operations were performed on four groups of CD1 male mice—sham/IgG1, sham/α-LOXL2, TAC/IgG1 and TAC/α-LOXL2 (Fig. 4a). The α-LOXL2 or control IgG1 treatment (intraperitoneally, 30 mg kg^−1^, twice per week) was initiated 2 weeks after TAC, when the heart already displayed 35% hypertrophy (by ventricle–body weight ratio) and 25% reduction of left ventricular fractional shortening (LVFS by echocardiography) (Fig. 4a). Remarkably, within 2 weeks of treatment, α-LOXL2 stabilized LVFS of TAC-stressed hearts, preventing further LVFS decline with a trend toward normalizing LVFS after 8 weeks of treatment (Fig. 4a). Ten weeks after TAC (8 weeks after treatment), the IgG1-treated group developed severe cardiac hypertrophy and fibrosis with 80.9% increase of ventricle–body weight ratio, 87.9% increase of end-systolic LV internal diameter (LVIDs), 39.0% increase of end-diastolic LV internal diameter (LVIDd), and 49.1% reduction of LVFS (Fig. 4b–g). Conversely, α-LOXL2-treated mice displayed much less TAC-induced cardiac dysfunction. The interstitial fibrosis was essentially eliminated, with LVFS increased by 50.9% (*P*=0.01) and LV dilatation reduced by 25.0% (LVIDd, Student's *t*-test, *P*=0.03; Fig. 4b–f). Notably, these major improvements of left ventricular function occurred without significant reduction of cardiomyocyte hypertrophy (measured by ventricle–body weight ratio; Fig. 4g) and in the absence of significant changes of immune cell infiltration into the myocardium (Supplementary Fig. 2a,b). This suggests that the beneficial effects of α-LOXL2 are caused not by attenuation of hypertrophy or immune-mediated cardiac damage but rather by α-LOXL2's reversal of interstitial fibrosis. Such anti-fibrosis effects of α-LOXL2 are likely the results of reduced collagen production and enhanced degradation of un-crosslinked collagen^15^. Collectively, the Loxl2 expression and inhibition data indicate that Loxl2 expression activated by cardiac stress can trigger interstitial fibrosis and cardiac dysfunction.

To assess how Loxl2 controlled ventricular function of stressed hearts, we used a micro-admittance catheter to measure left ventricular pressure–volume (PV) relationships of anaesthetized mice *in vivo*. Analyses of mice after 10 weeks of TAC with 8 weeks of α-LOXL2 treatment showed that LV pressure overload was comparable between the control IgG and α-LOXL2-treated hearts (Supplementary Fig. 2c), but α-LOXL2 dramatically reduced the left ventricular size and end-diastolic pressure (EDP) of stressed hearts (Fig. 4h). α-LOXL2 improved ejection fraction by 107% (Student's *t*-test, *P*<0.01), stroke volume by 73% (Student's *t*-test, *P*=0.01), stroke work by 48% (Student's *t*-test, *P*=0.01), and preload-adjusted maximal power (plPwr) by 78% (Student's *t*-test, *P*=0.01; Fig. 4i–l). Strikingly, the stroke work and plPwr were normalized (Fig. 4k,l), indicating that Loxl2 inhibition prevents stress-induced systolic abnormalities and restores cardiac contractile function to the sham control level.

Consistent with the recovery of cardiac contractile work, α-LOXL2 reduced the left ventricular end-systolic volume (ESV) by 43% (Student's *t*-test, *P*<0.001) and end-diastolic volume (EDV) by 19% (Student's *t*-test, *P*<0.01) (Fig. 4m,n). This led to the elimination of TAC-induced enlargement of heart chamber at end diastole, reducing the heart size to the sham control level (Fig. 4n).

Of note, the pressure overload by itself—without invoking cardiomyopathy—is sufficient to increase ESV (but not EDV) of a healthy heart, thus leading to a myopathy-independent, mathematical reduction of EF, which equals (1 minus ESV/EDV). Given this pressure effect, we concluded that the residual ESV and EF changes of α-LOXL2-treated hearts are primarily the result of persistent pressure load. This conclusion is supported by the normalization of EDV, stroke work and contractile power of α-LOXL2-treated hearts. Therefore, α-LOXL2 is capable of decreasing stress-related cardiac dilatation and cardiac contractile dysfunction.

In addition, α-LOXL2 normalized the diastolic function of stressed hearts. In TAC-stressed hearts, myocardial relaxation was impaired, and the left ventricles became stiff. The myocardial relaxation abnormalities were evident by an increase of isovolumic relaxation time constant (Tau, 2.32-fold increase) and decline of diastolic pressure relaxation (maximal diastolic dp/dt, 24% reduction; Fig. 4o,p). Besides abnormal relaxation, the TAC-stressed left ventricles were stiff, as shown by the steep slopes of diastolic pressure–volume curves of stressed hearts (Fig. 4q). The combination of slow relaxation and stiff ventricles resulted in elevated left ventricular filling pressure or end-diastolic pressure (EDP), 3.83-fold over the control level (Fig. 4r). Conversely, in α-LOXL2-treated, TAC-stressed hearts, Tau was reduced by 81% (Student's *t*-test, *P*=0.01) to levels not significantly different from the controls (Fig. 4o). Also, EDP was reduced by 74% (Student's *t*-test, *P*<0.01), with near normalization to the control level (Fig. 4r). Therefore, the TAC-stressed hearts treated with α-LOXL2 had minimal fibrosis (Fig. 4b,c) and compliant ventricles with essentially normal filling pressure. The data indicate that Loxl2 is essential for the development of interstitial fibrosis, ventricular stiffness and diastolic dysfunction in pathologically stressed hearts.

The combined effects of α-LOXL2 on the systolic and diastolic abnormalities resulted in 70% enhancement of cardiac output (Student's *t*-test, *P*=0.01; Fig. 4s) and normalization of serum levels of cardiac stress marker (BNP) and fibrosis marker (TIMP-1; Fig. 4t,u). Moreover, α-LOXL2-treated mice showed a mortality rate of 20% over 30 weeks of observation, whereas IgG-treated TAC mice had 70% mortality (Fig. 4v). Therefore, α-LOXL2 is effective in protecting the hearts from stress-induced failure.

### Loxl2 knockout protects stress-induced cardiac dysfunction

Next we asked whether Loxl2, among all Lox isoforms, played a specific role in HF pathogenesis. To address that, we used CRISPR/Cas9 and two-cut strategy to generate LoxP-flanked (floxed) alleles of the mouse *Loxl2* gene (*Loxl2*^*fl/fl*^) and then used the Cre-Lox and tamoxifen-induction methods to exert genetic knockout of *Loxl2* at the desired time window (Supplementary Fig. 3a). The LoxP sequences that flanked exon 8 and 9 of *Loxl2*, once recognized and recombined by Cre recombinase, would enable the deletion of the two exons, resulting in disruption of gene regions encoding the SRCR and downstream catalytic domains. The SRCR domain is the Loxl2 region targeted by anti-LOXL2 (ref. 16; Supplementary Fig. 3a). The floxed alleles were genotyped by PCR and confirmed by sequencing (Supplementary Fig. 3b,c). To delete *Loxl2*, we crossed *Loxl2*^*fl/fl*^ mice to a driver mouse line that carried the transgene *ActinCre*^*ERT*^. The expression of *ActinCre*^*ERT*^ is driven by the chicken beta-actin promoter/enhancer coupled with the cytomegalovirus immediate-early enhancer^17^, and its Cre recombinase (Cre^ERT^) requires tamoxifen to activate the enzymatic function. The *ActinCre*^*ERT*^*; Loxl2*^*fl/fl*^ genetic combination, therefore, allowed us to use tamoxifen to induce global *Loxl2* knockout in adult mice, mimicking the systemic administration of anti-LOXL2. We treated the mice with tamoxifen for 5 days to delete *Loxl2* exon 8 and 9 from the genome (Supplementary Fig. 3d). Interestingly, the *Loxl2* mRNA that lacked exon 8 and 9 became destabilized and could not be detected by qPCR primers that targeted either deleted or non-deleted regions of the mRNA (Supplementary Fig. 3e). Neither could fragments of Loxl2 proteins be detected by western blotting using polyclonal antibodies (Supplementary Fig. 3f). These findings indicate a complete loss of Loxl2 protein in the knockout mice.

**Figure 5:**
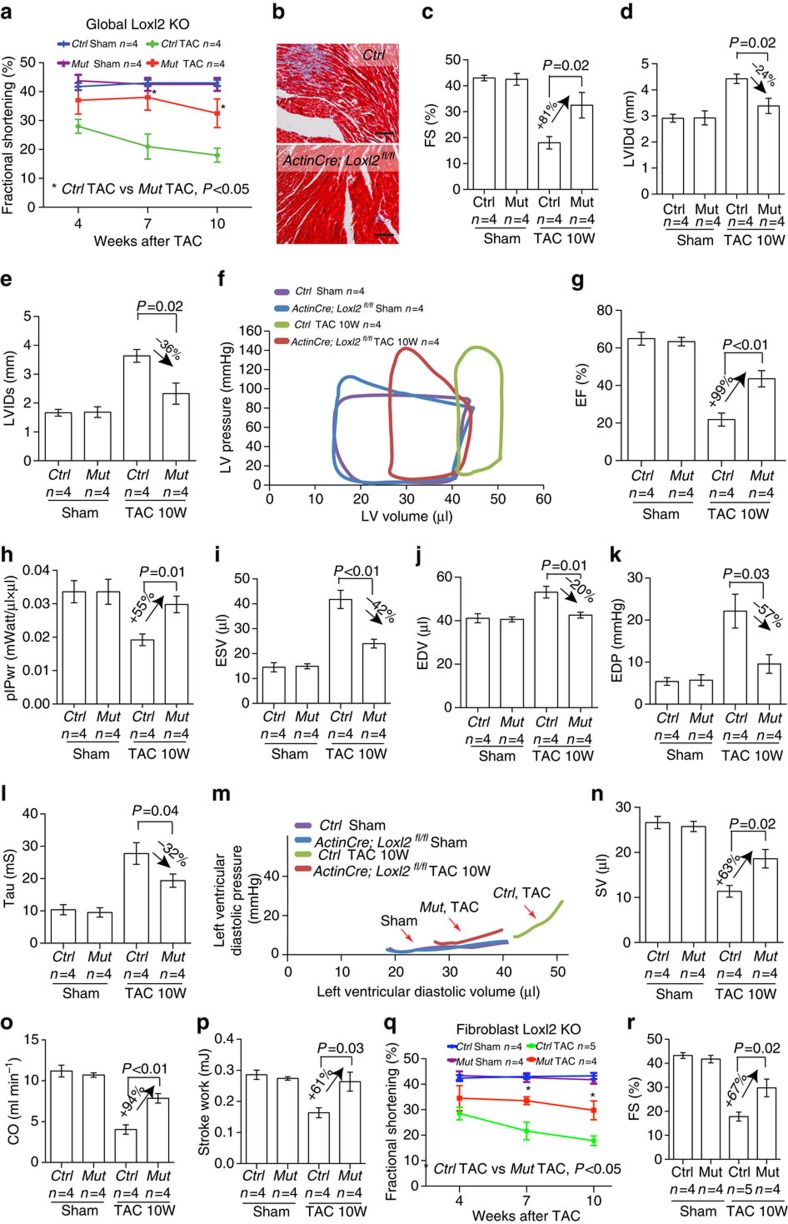
LOXL2 gene deletion improves cardiac function of stressed hearts in mice. (**a**) Cardiac fractional shortening changes of *ActinCre*^*ERT*^*;Loxl2*^*fl/fl*^ KO mice over 10 weeks (10W) after TAC. Ctrl: *ActinCre*^*ERT*^*; Loxl2*^*fl/+*^, *Loxl2*^*fl/fl*^ or *Loxl2*^*fl/+*^. KO: *ActinCre*^*ERT*^*; Loxl2*^*fl/fl*^. (**b**) Cardiac interstitial fibrosis detected by Masson's trichrome staining in Ctrl or Loxl2-null mice 10 weeks after sham or TAC operation. Scale bars, 100 μm. Red: cardiomyocytes. Blue: fibrosis. (blue: collagen staining). (**c**–**e**) Echocardiographic measurement of left ventricular fractional shortening (**c**), left ventricular internal diameter at diastole (LVIDd, **d**) and at systole (LVIDs, **e**) after 10 weeks of TAC. *P* value: Student's *t*-test. Error bar: s.e.m. (**f**) Representative cardiac PV-Loop of *Ctrl* or *Loxl2*-null mice 10 weeks after sham or TAC operation. (**g**–**p**) Quantification of ejection fraction (**g**), preload-adjusted maximal power (plPwr, **h**), end-systolic volume (**i**), end-diastolic volume (**j**), end-diastolic pressure (**k**), isovolumic relaxation time constant Tau (**l**), diastolic pressure–volume relationships (**m**), stroke volume (**n**), cardiac output (**o**) and stroke work (**p**) of *Ctrl* or *Loxl2*-null mice 10 weeks after sham or TAC operation. *P* value: Student's *t*-test. Error bar: s.e.m. (**q**,**r**) Cardiac fractional shortening changes of *iTcf21Cre*^*ERT*^*; Loxl2*^*fl/fl*^ mutant mice over 10 weeks after TAC (**q**), and quantification of fractional shortening at 10 weeks after TAC (**r**). *Ctrl*: *iTcf21Cre*^*ERT*^*; Loxl2*^*fl/+*^, *Loxl*^*fl/fl*^ or *Loxl*^*fl/+*^. *Mut*: *iTcf21Cre*^*ERT*^*; Loxl2*^*fl/fl*^. *P* value: Student's *t*-test. Error bar: s.e.m.

Male mice of Loxl2-null and their littermate control (*ActinCre*^*ERT*^*; Loxl2*^*fl/+*^ or *Loxl2*^*fl/fl*^, *Loxl2*^*fl/+*^) at 8–10 weeks of age were then subjected to TAC and followed for the development of HF for 10 weeks (Fig. 5a). During the course, there were no observable adverse effects of *Loxl2* mutations on the cardiac or gross functions of the mice, suggesting that *Loxl2* is dispensable for the baseline function of mice. However, Loxl2 knockout prevented TAC-induced cardiac interstitial fibrosis and dysfunction (Fig. 5b–e), but no effects on myocyte hypertrophy (Supplementary Fig. 4a). Interstitial fibrosis was essentially eliminated (Fig. 5b), with 81% increase of cardiac FS (Fig. 5c) and 24–36% reduction of LV dimensions measured by echocardiography (Fig. 5d,e). Cardiac catheterization and PV loop analysis further confirmed that Loxl2 deletion protected the heart from TAC-induced chamber dilatation and functional decline. After 10 weeks of TAC, the *Loxl2*-null mice (*ActinCre*^*ERT*^*; Loxl2*^*fl/fl*^) exhibited great improvement of cardiac mechanical function (Fig. 5f). In Loxl2-null mice, the systolic function was greatly improved: Cardiac EF was improved by 99%, plPwr increased by 55% (normalized), ESV reduced by 42% and EDV reduced by 63% (normalized; Fig. 5g–j). The diastolic function was also greatly improved. EDP was reduced by 57%, Tau reduced by 32% and the slope of diastolic PV curve reduced, approaching the diastolic slope of compliant hearts (Fig. 5k–m). Overall, the cardiac performance was greatly improved by anti-LOXL2: SV increased by 63%, CO by 94% and SW by 61% (normalized; Fig. 5n–p). Consistently, cardiac stress and fibrosis gene markers—*β-MHC*, *β/α-MHC ratio*, *Bnp, Timp-1*, *α-SMA*, *Col1A*, *Col3A*, *Opn* and *Osf2*—were much reduced and essentially normalized in *Loxl2*-null hearts (Supplementary Fig. 4b,c). Taken together, the genetic studies thus demonstrate a requirement of Loxl2 in the development of HF.

To map the cellular site where Loxl2 functioned to trigger HF, we performed tissue-specific knockout of Loxl2 in cardiac fibroblasts, using the promoter of *Tcf21*—a transcription factor specifically expressed in fibroblasts^18^—to drive the expression of Cre recombinase (*Cre*^*ERT*^) whose enzymatic activity is inducible by tamoxifen (*iTcf21Cre*^*ERT*^; Supplementary Fig.5a,b). Fibroblast-specific deletion of *Loxl2* can therefore be executed in *iTcf21Cre*^*ERT*^*;Loxl2*^*fl/fl*^ mice treated with tamoxifen (Supplementary Fig. 5c). By the end of 10 weeks after TAC, mice without Loxl2 in cardiac fibroblasts exhibited fractional shortening (FS) 67% higher than that of mice expressing Loxl2 in fibroblasts (Fig. 5q,r). The magnitude of FS improvement in mice lacking fibroblast Loxl2 is comparable to that of anti-LOXL2 therapy and to that of global Loxl2 knockout at the corresponding time window (Figs 4a and 5a). These observations indicate a crucial role of Loxl2 secreted by fibroblasts in HF pathogenesis.

### LOXL2 promotes TGF-β2 production through the PI3K path

**Figure 6:**
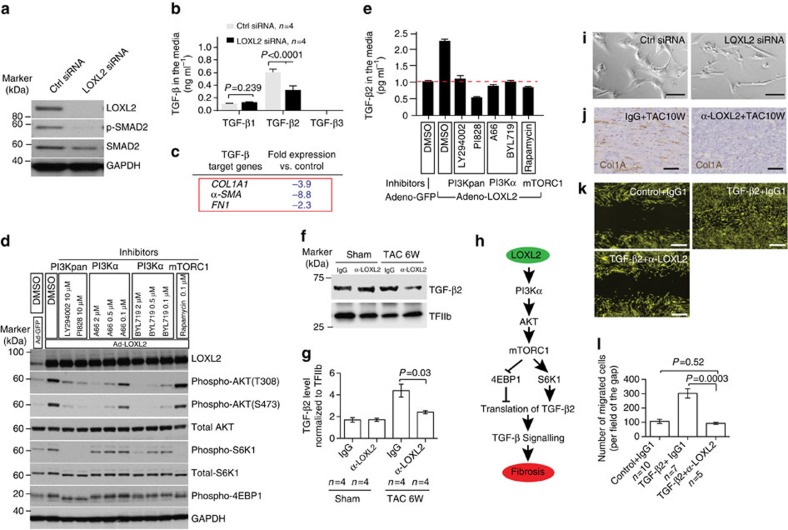
LOXL2 acts with TGF-β through PI3K/ATK/mTORC1 signalling to control myofibroblast transformation and migration. (**a**) Western blot analysis of LOXL2, SMAD2, p-SMAD2 and GAPDH in human primary cardiac fibroblasts transfected with control (*Ctrl*) or *LOXL2* siRNA. (**b**) Quantification of TGF-β isoforms in the culture media of human primary cardiac fibroblasts transfected with control or *LOXL2* siRNA (*n*=4). *P* value: Student's *t*-test. Error bar: s.e.m. (**c**) Changes of *COLA1*, *α-SMA* and *FN1* mRNA in human primary cardiac fibroblasts transfected with control (Ctrl) or *LOXL2* siRNA. (**d**) Western blot of LOXL2, p-AKT, AKT, p-S6K, S6K and p-4E-BP1 with or without PI3K inhibitors LY294002/PI828 (10 μM each), PI3Kα inhibitors A66/BYL719 and mTORC1 inhibitor Rapamycine (0.1 μM) in cells infected with Ad_GFP/Ad_LOXL2. (**e**) TGF-β2 protein in the culture media (*n*=4). *P* value: Student's *t*-test. Error bar: s.e.m. (**f**,**g**) Western blot (**f**) and quantification (**g**) of TGF-β2 protein in the mice heart ventricles 6 weeks after sham/TAC operation. *n*=4 mice per group. *P* value: Student's *t*-test. Error bar: s.e.m. (**h**) A signalling cascade from LOXL2 to PI3K/AKT/mTORC1 to TGF-β2 translation. (**i**) Representative phase-contrast image of human primary cardiac fibroblasts 72 h after transfection with control (Ctrl) or *LOXL2* siRNA. Scale bars, 50 μm. (**j**) Immunostaining of Col1A in IgG1- or α-LOXL2-treated mice 10 weeks (10W) after sham or TAC operation. Scale bars, 100 μm. Blue: haematoxylin. Brown: Col1A. (**k**,**l**) Gap closure assay of fibroblast migration in control, TGF-β2, α-LOXL2 (**k**) groups and quantification of cells migrating into the gap (**l**). Scale bars, 200 μm. *n*=5–10, *P* value: Student's *t*-test. Error bar: s.e.m.

Although α-LOXL2 and LOXL2 specific gene deletion essentially eliminated interstitial fibrosis and ventricular stiffness, it had only marginal effect on cardiomyocyte hypertrophy (Fig. 4g and Supplementary Fig. 4a). The uncoupling of myocyte hypertrophy and interstitial fibrosis suggests that LOXL2 provides a myocyte-independent, interstitial mechanism to control stress-induced matrix modifications. In the interstitium of stressed hearts, TGF-β provides the primary signal to trigger the transdifferentiation of fibroblasts into myofibroblasts, which then migrate to and deposit collagen in stressed areas of the heart^4^,^5^,^15^,^19^,^20^,^21^,^22^,^23^,^24^,^25^,^26^. To test whether LOXL2 was required for TGF-β-induced cardiac myofibroblast transformation, we knocked down *LOXL2* and examined TGF-β activity in the primary human cardiac ventricular fibroblasts. Human cardiac fibroblasts contained abundant LOXL2, and *LOXL2*-targeted siRNA effectively removed LOXL2 from these cells (Fig. 6a). In LOXL2-deficient cells, the phosphorylation of SMAD2 was nearly absent without significant changes of SMAD2 protein amount (Fig. 6a), suggesting inactive TGF-β signalling. Quantification of TGF-β ligands revealed that the human cardiac fibroblasts secreted primarily TGF-β2 and that LOXL2 was required for TGF-β2 production. Without LOXL2, the TGF-β2 ligand secreted by fibroblasts was reduced by ∼50% (Student's *t*-test, *P*<0.0001; Fig. 6b), accompanied by reduced expression of TGF-β target genes, *COL1A*, *FN1* and *α-SMA* (Fig. 6c) that are characteristic markers of myofibroblasts^27^. Consistent with the results from *LOXL2* knockdown experiments, overexpression of *LOXL2* in cardiac fibroblasts increased TGF-β2, but not TGF-β1 or TGF-β3 production in the culture media (Supplementary Fig. 6a). Interestingly, LOXL2 increased TGF-β2 protein production without significant effect on TGF-β2 mRNA expression (Supplementary Fig. 6b), suggesting that LOXL2 regulates TGF-β2 at the translation level. Given that the PI3K/AKT/mTORC1 signalling pathway is known to regulate translation and promote heart failure^28^,^29^, we tested whether LOXL2 functioned through the PI3K path to enhance TGF-β2 translation. We found that LOXL2 overexpression in human cardiac fibroblasts increased AKT phosphorylation at serine 473 and threonine 308, without changing total AKT amount (Fig. 6d). LOXL2 also increased phosphorylation of the mTORC1 target protein S6K and 4E-BP1 without changing their total protein levels (Fig. 6d). Notably, PI3Kα (but not PI3β, PI3Kγ or PI3Kδ) knockdown or inhibition decreased LOXL2-induced AKT/mTORC1 signalling and reduced TGF-β2 protein level in the culture media (Supplementary Fig. 6c–f). These results suggested that LOXL2 activates PI3K/AKT/mTORC1 signalling in cardiac fibroblasts. Such PI3K activation by LOXL2 was required for TGF-β2 production. PI3K and mTORC1 inhibition not only blocked TGF-β2 signalling activation (Fig. 6d), but also prevented the LOXL2-induced TGF-β2 production (Fig. 6e).

We also measured TGF-β2 level in heart tissues by western blotting after 6 weeks of TAC with or without antibody treatment. We found that cardiac TGF-β2 level increased by ∼2.5-folds in TAC-stressed hearts, whereas in α-LOXL2-treated TAC hearts, such stress-induced change of TGF-β2 was reduced by 55% (Student's *t*-test, *P*=0.03) to levels not significantly different from the controls (Fig. 6f,g). Consistent with the antibody treatment data, in TAC-stressed *Loxl2*-null hearts, the target genes of TGF-β—*α-SMA*, *Col1A* and *Col3A*—were reduced to normal level (Supplementary Fig. 4c). These *in vivo* data are consistent with the ability of Loxl2 to stimulate production of TGF-β2 in fibroblasts. Collectively, these results indicate a signalling cascade from LOXL2 to PI3Kα/AKT/mTORC1 and then to TGF-β2 to stimulate cardiac fibrosis (Fig. 6h).

### LOXL2 controls myofibroblast transformation and migration

The regulation of myofibroblast marker expression by LOXL2 suggested that LOXL2 was required for fibroblasts to transform into myofibroblasts. To test that, we examined fibroblast cell morphology in cell culture. Myofibroblasts are morphologically large and polygonal, whereas fibroblasts are slender and spindle-shaped^30^. Under cell culture conditions, human cardiac fibroblasts produced TGF-β2 and displayed morphological features of the large, polygonal myofibroblasts (Fig. 6i). In contrast, LOXL2-deficient fibroblasts, with reduced TGF-β signalling and *α-SMA* expression (Fig. 6b,c), were slender and spindle-shaped (Fig. 6i), consistent with a lack of myofibroblast transformation. Therefore, both the marker and cell morphology studies indicate that LOXL2 promotes the formation of cardiac myofibroblasts, which are critical for the development of ventricular fibrosis^2^,^4^,^5^. This conclusion is further supported by the observation that LOXL2 inhibition nearly eradicated myofibroblast-dependent Col1A deposition and fibrosis in TAC-stressed mouse hearts (Figs 4b and 6j).

**Figure 7:**
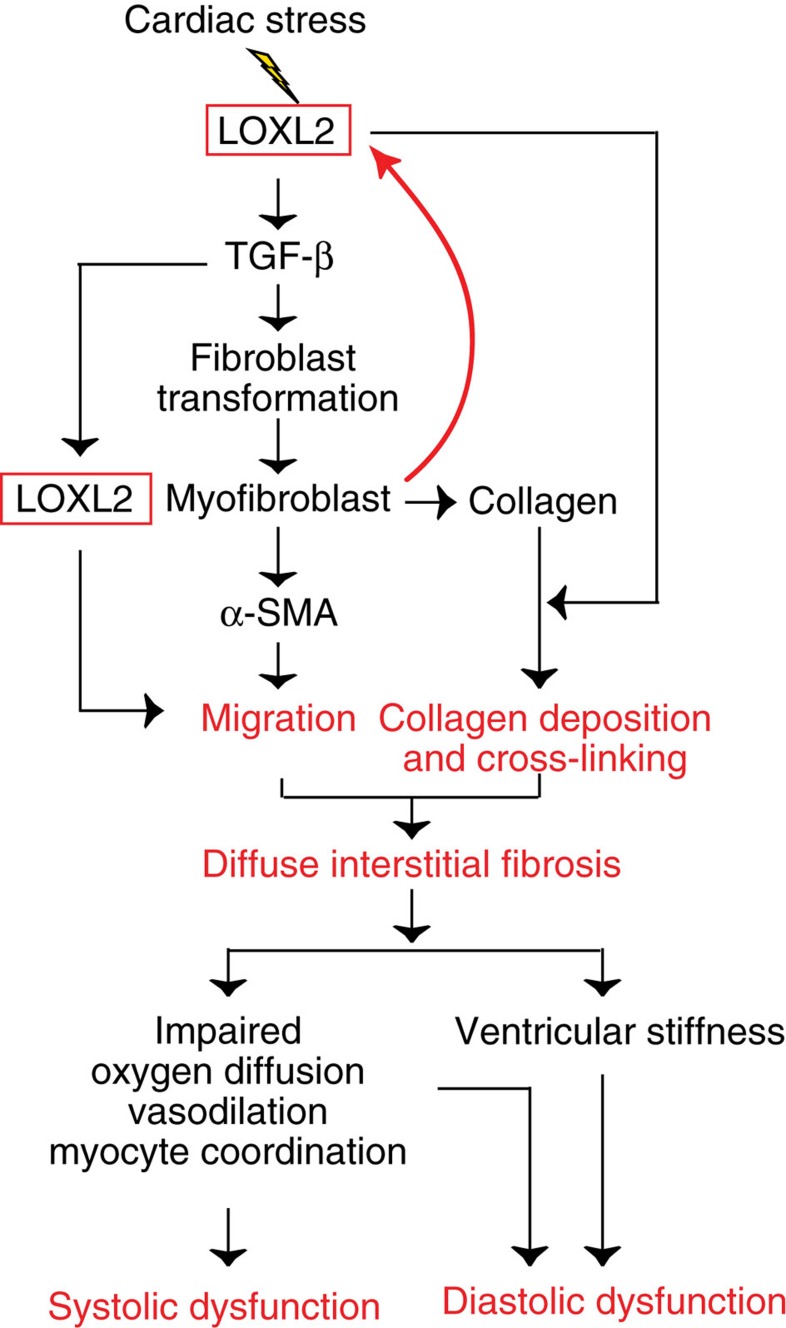
Working model. A working model of how cardiac stress activates LOXL2 to trigger myofibroblast transformation, collagen synthesis, collagen crosslinking and myofibroblast migration, leading to diffuse ventricular fibrosis and dysfunction.

In the stressed hearts, the newly formed myofibroblasts migrate to the stressed or hypertrophic region, where the myofibroblasts produce collagen-rich matrix, causing ventricular fibrosis^2^,^4^,^5^. Given that myofibroblast migration requires TGF-β signals^27^,^31^,^32^,^33^, we tested whether LOXL2 was essential for controlling myofibroblast migration using a gap closure assay. Human ventricular fibroblasts were seeded onto collagen I-coated plates, and the cell monolayer was scratched to create a cell-free zone or gap on the plate (Fig. 6k). The cells were then treated with TGF-β2 to stimulate cell migration into the gap (Fig. 6k). In this assay, TGF-β2 enhanced the number of cells migrating into the gap by ∼3-fold, whereas α-LOXL2 completely blocked such effect of TGF-β2 (Fig. 6l). Because TGF-β2 was added exogenously in the assay, the inhibition of cell migration by α-LOXL2 was not a result of inadequate TGF-β2 secretion (Fig. 6b). LOXL2 inhibition likely changed collagen crosslinking and the microstructure of the extracellular matrix to inhibit fibroblast migration. Collectively, the *in vitro* and *in vivo* studies indicate that LOXL2 is a crucial molecule activated by cardiac stress to transform and mobilize fibroblasts, triggering collagen synthesis, crosslinking and myofibroblast migration, thereby causing diffuse interstitial fibrosis and ventricular dysfunction (Fig. 7).

## Discussion

Cardiac interstitial fibrosis is a major cause of systolic and diastolic abnormalities^2^,^3^,^4^,^5^,^6^ and a strong predictor of the clinical outcomes of patients with HF across a wide spectrum of disease severity^6^. However, the fibrotic process has not been a direct therapeutic target for HF. In patients with HF, LOXL2 levels are elevated in heart tissues and serum, its levels correlating with cardiac dysfunction and HF biomarker levels. In mice, LOXL2 activation is essential for cardiac fibrosis and HF development. The genetic or pharmacological inhibition of LOXL2 greatly reduces cardiac fibrosis and halts HF progression. The human sample and animal efficacy studies, in combination, suggest a pathogenic role of LOXL2 in cardiac fibrosis and human HF. Our studies therefore delineate a novel LOXL2-mediated HF mechanism and provide new insights into HF therapy.

In mechanically stressed hearts, LOXL2 expression is activated in the fibroblasts, which then release LOXL2 proteins into the interstitial space, particularly in the hypertrophic area where the mechanical stress is highest (Fig. 7). This suggests that LOXL2 activation is a response of cardiac fibroblasts to enhanced mechanical stress to maintain structural integrity of the heart. Indeed, LOXL2 elevation has multiple biological effects that promote interstitial collagen formation. LOXL2 not only triggers myofibroblast transformation to enhance collagen production (through PI3K-AKT-mTOR and TGF-β), but also augments collagen strength (by crosslinking collagen fibres). LOXL2 also stimulates myofibroblasts migration to large areas of the heart, where these cells produce collagen fibres. Collagen fibres, once released into the interstitial space, are crosslinked by LOXL2 to form bundles of collagen that are much stiffer than isolated collagen fibres. Furthermore, activated fibroblasts secrete more LOXL2 and collagen, creating a positive feedback loop to sustain the fibrotic process. All these factors, in combination, trigger diffuse interstitial fibrosis of pathologically stressed hearts.

The LOXL2-mediated stress reaction, although capable of reinforcing tissue strength in the face of increased mechanical stress, is ultimately maladaptive. Excessive amount of collagen fibres in cardiac interstitial space impede coronary vasodilation, oxygen diffusion and electromechanical coordination between cardiomyocytes, causing contractile abnormalities (systolic pump dysfunction)^34^. Meanwhile, the increase of collagen and its crosslinking stiffens the left ventricle, impairing ventricular relaxation and filling (diastolic pump dysfunction)^35^,^36^. Therefore, the LOXL2-mediated interstitial reaction leads to both systolic and diastolic abnormalities, revealing a novel therapeutic avenue for HF. The diastolic aspect of LOXL2 effects is particularly important, given the recognition and increasing prevalence of diastolic dysfunction as part of HF syndrome and the lack of approved therapy for HFpEF with primarily diastolic failure.

Although LOXL2 can interact with TGF-β in models of cancer and bone remodelling^14^,^37^, the roles of *LOXL2* gene in HF have not been demonstrated in mouse genetic models *in vivo*. By generating a new LOXL2 genetic model with fibroblast-specific LOXL2 knockout, we showed crucial roles of LOXL2 in cardiac fibroblasts for stress-induced interstitial fibrosis and cardiac dysfunction. What remains unanswered is how LOXL2-mediated changes of extracellular collagen composition affect TGF-β processing and signalling and guide the cellular process of myofibroblast migration. Given that LOXL2 is transcriptionally activated in the stressed hearts, it will be essential to know what factors control the expression of *LOXL2* gene in the hearts. Gene expression regulation can occur at the chromatin, transcription or posttranscriptional level, mediated respectively by epigenetic factors (chromatin-regulating factors, long noncoding RNAs), transcription factors and microRNAs. Understanding LOXL2 regulation will provide an opportunity to integrate cardiac fibrosis with epigenetics and RNA mechanisms of HF and to identify additional new targets for HF therapy.

## Methods

### Animal sample size and randomization

Littermate CD1 male mice were purchased from Charles River (Strain Code: 022). *ActinCre*^*ERT*^ (ref. 17) and *Rosa-mT/mG* (ref. 38) mice were purchased from Jackson Lab. *iTcf21Cre*^*ERT*^ (ref. 18) was provided by Dr Michelle D. Tallquist at the University of Hawaii and Dr Eric Olson at the UT Southwestern. *Loxl2*^*fl/+*^ mice were generated by the Chang Lab. The number of animals used (*n*) was denoted in each test in the figures, including technical replicates when applicable. Each subgroup of experiments had *n*=8 to 10 biological replicates, many of which had technical replicates of three. The mice were randomly selected from the cage and assigned to different control and experimental subgroups as described in the text. The control and experimental groups are blinded to the operators of echocardiography, catheterization and heart tissue analyses. The use of mice for studies was in compliance with the regulations of Indiana University and National Institute of Health.

### Study population and analysis of human heart samples

The patients presenting at the Charité University Medicine Berlin with heart failure symptoms and reduced exercise capacity despite preserved LVEF (>50%; heart failure with preserved ejection fraction, HFpEF) who showed diastolic dysfunction according to echocardiographic analysis and European Society of Cardiology recommendations^13^ were enrolled in the study (*n*=24) (Table 2). All the patients were evaluated by echocardiography, invasive angiography, 6-min walk test and NT-proBNP. The patients without symptoms and signs of congestive heart failure but with atypical angina or intermittent arrhythmias who underwent right ventricular (RV) biopsy for evaluation of cardiomyopathy and showed regular systolic and diastolic LV function were enrolled as controls (*n*=15; Table 2). Endomyocardial biopsies were obtained from the RV septum as previously described^35^. Collagen volume fraction was analysed with collagen-specific Picrosirius red. Crosslinking was calculated as the ratio of insoluble to soluble collagen forms by a colorimetric approach in nine control subjects and 14 HFpEF patients^39^. Immunohistochemical staining was performed in heart tissues of adequate quality and quantity^35^. Other heart tissue samples used for LOXL2 immunostaining and for *LOXL2*, *COL1A* and *COL3A* mRNA quantification were derived from patients with ischaemic or idiopathic dilated cardiomyopathy and from heart transplantation donors that did not have heart failure (Table 1). The use of human subjects or tissue samples was in compliance with the regulations of Charité University, Gilead Sciences and Indiana University.

### Biomarker analysis in heart failure patients

Serum LOXL2 was measured using customized RUO LOXL2 kits (bioMérieux) on Vitek Immuno Diagnostic Assay System (VIDAS) platform. The plasma concentrations of ST-2 and tissue inhibitor of metalloproteinase-1 (TIMP-1) were determined using ELISA kits obtained from R&D Systems and NT-proBNP was measured using a Luminex kit from EMD Millipore following manufacturers' instructions. The serum level of cardiac troponin I was measured using Simoa cTnI assay (Quanterix Corp).

### Antibody production and purification

Hybridoma cells expressing anti-LOXL2 (AB0023) antibody were transferred to Aragen Bioscience (Gilroy, CA, USA) for the production of ascites fluid in BALB/c mice. Ascites fluid was then purified by batch mode on MabSelect resin and dialysed into phosphate-buffered saline (PBS) with 0.01% Tween 20. Purified AB0023 was then tested in multiple assays for release. The control IgG1 antibody directed against a non-naturally occurring protein was purchased from Antibody Solutions (Sunnyvale, CA, USA).

### Transaortic constriction

Surgeries were adapted from ref. 40 and were performed on CD1 male mice of 6–8 weeks of age, and Loxl2 genetic male mice of 8–10 weeks of age and between 20 and 25 grams of weight. The mice were anaesthetized with isoflurane (2–3%, inhalation) in an induction chamber and then intubated with a 20-gauge intravenous catheter and ventilated with a mouse ventilator (Minivent, Harvard Apparatus, Inc). Anaesthesia was maintained with inhaled isoflurane (1–2%). A longitudinal 5-mm incision of the skin was made with scissors at midline of sternum. The chest cavity was opened by a small incision at the level of the second intercostal space 2–3 mm from the left sternal border. The chest retractor was gently inserted to spread the wound 4–5 mm in width. The transverse portion of the aorta was bluntly dissected with curved forceps. Then, 6-0 silk was brought underneath the transverse aorta between the left common carotid artery and the brachiocephalic trunk. One 26-gauge needle was placed directly above and parallel to the aorta. A loop was then tied around the aorta and needle, and secured with a second knot. The needle was immediately removed to create a lumen with a fixed stenotic diameter. The chest cavity was closed by 6-0 silk suture. The sham-operated mice underwent similar surgical procedures, including isolation of the aorta, looping of aorta, but without tying of the suture. The pressure load caused by TAC was verified by the pressure gradient across the aortic constriction measured by echocardiography. Only mice with a peak pressure gradient >30 mm Hg were analysed for cardiac hypertrophy and gene expression.

### Echocardiography

The echocardiographer was blinded to the genotypes, surgical or pharmacological treatment of the mice tested. Transthoracic ultrasonography with a GE Vivid 9 ultrasound platform (GE Health Care, Milwaukee, WI, USA) and a 13 MHz transducer was used to measure aortic pressure gradient and left ventricular function. To minimize the confounding influence of different heart rates on aortic pressure gradient and left ventricular function, the flow of isoflurane (inhalational) was adjusted to anaesthetize the mice while maintaining their heart rates at 450–550 beats per minute. The peak aortic pressure gradient was measured by continuous wave Doppler across the aortic constriction. The left ventricular function was assessed by the M-mode scanning of the left ventricular chamber, standardized by two-dimensional, short-axis views of the left ventricle at the mid papillary muscle level. The fractional shortening (FS) of the left ventricle was defined as 100% × (1−end-systolic/end-diastolic diameter), representing the relative change of left ventricular diameters during the cardiac cycle. The mean FS of the left ventricle was determined by the average of FS measurement of the left ventricular contraction over five beats. The *P* values were calculated by the Student's-*t* test. The error bars indicate standard error of mean.

### *In vivo* catheterization

The trachea was exposed by a midline incision from the base of the throat to just above the clavicle. The mice were intubated with a piece of polyethylene-90 tube. After the tube was secured in place by using a 6-0 silk suture, 100% oxygen was gently blown across the opening. The mice receiving ventilation were placed on a warmed (37 °C) pad. The right carotid artery was then isolated. Care was taken to prevent damage to the vagal nerve. The mice were lightly anaesthetized with isoflurane maintaining their heart rates at 450–550 beats per minute. A 1.2F Pressure-Volume Catheter (FTE-1212B-4518, Scisense, Inc) was inserted into the right carotid artery and then advanced into the left ventricle. The transducer was securely tied into place, after it was advanced to the ventricular chamber as evidenced by a change in pressure curves. The hemodynamic parameters were then recorded in close-chest mode. The parameters include left ventricular systolic pressure, EF, plPwr, stroke volume, stroke work, ESV, EDV, Tau, EDP and cardiac output.

### Generation of Loxl2-floxed mice by *CRISPR*/*Cas9* gene editing

Two sgRNAs (5′sgRNA and 3′sgRNA) were designed to target the Loxl2 locus (the intron between exon 7 and 8 and the intron between exon 9 and 10; illustrated in Supplementary Fig. 3) using online software (http://tools.genome-engineering.org)^41^. T7 promoter was added to the sgRNA templates by PCR amplification, using PX330 as template with primer sets Loxl2-5′LoxP-sg-F and T7-sgRNA Common_R, and Loxl2-3′LoxP-sg-F and T7-sgRNA Common_R. SgRNA sequences are underlined in the primers.

Loxl2-5′LoxP-sg-F: 5′-TTAATACGACTCACTATAGGGGGTCCTAGATGGCCACCCT GTTTTAGAGCTAGAAATAGCAAGTT-3′

Loxl2-3′LoxP-sg-F: 5′-TTAATACGACTCACTATAGGCTCGGGGTGGGGAAGCGCCG GTTTTAGAGCTAGAAATAGCAAGTT-3′

T7-sgRNA Common_R: 5′-AAAAGCACCGACTCGGTGCC-3′

The T7-sgRNA PCR product was gel purified and used as the template for *in vitro* transcription using MEGAshortscript T7 kit (Life Technologies).

We first tested the cleavage efficiency of sgRNAs at the genomic target site. Capped polyadenylated Cas9 mRNA (Sigma-Aldrich; 100 ng ml^−1^) was co-injected with sgRNAs (50 ng ml^−1^) into pronuclear (PN) stage one-cell mouse embryos and assessed the frequency of altered alleles (insertions and deletions) at the blastocyst stage using PCR assay with following primer sets.

Loxl2-5′sg-test F: 5′-ACCTATGAGTGAATGACCCCTG-3′

Loxl2 5′sg-test R: 5′-GGTCCAAGAAAGGTATAACGG-3′

Loxl2 3′sg-test F: 5′-GTTTCACAGTGTGTGCTCGG-3′

Loxl2 3′sg-test R: 5′-ACTAATACACACTCACTCCC-3′

Both sgRNAs achieved over 50% indel at the genomic target site. Plasmid-based donor repair templates that contain homology arms (980 bp length on each side) and 5′ and 3′ *LoxP* sequences at denoted sites (Supplementary Fig. 3) were cloned to the TOPO-Blunt vector (Thermo Fisher) and verified by sequencing. Cas9 mRNA (100 ng ml^−1^), 5′-sgRNA (50 ng ml^−1^), 3′-sgRNA (50 ng ml^−1^) and donor circular dsDNA (100 ng ml^−1^) were mixed and injected into the cytoplasm of fertilized eggs with well-recognized pronuclei in the M2 media (Sigma-Aldrich). Injected zygotes were cultured in KSOM (media for *in vitro* embryo culture, MR-121-D, EMD Millipore) with amino acids at 37°C and 5% CO_2_ after the two-cell stage and were then transplanted to pseudopregnant CD1 females. Three out of 23 mice were genotyped positive with 5′ and 3′ *LoxP* insertion using the upstream PCR primer set 1 (product size 1,069 bp) and the downstream primer set 2 (product size 1,082 bp).

Primer set 1 (upstream)

5′ Loxl2-G F: 5′-CCTTCTGGAACTGCCAGAAGTATTTTAAG-3′ (upstream of template DNA sequence)

5′ Loxl2-G R: 5′-CGTATAATGTATGCTATACGAAGTTATTTGGATTCAGAACTTCC-3′

Primer set 2 (downstream)

3′ Loxl2-G F: 5′-GTATAGCATACATTATACGAAGTTATCCGTGGATTCTGGG-3′

3′ Loxl2-G R: 5′-CAGAGAGAAGATCCCCATCAG-3′ (downstream of template DNA sequence).

These three founder mice were grossly normal and heterozygous as determined by additional PCR primer sets—Loxl2-5′sg-test F and R (primer set 3), as well as Loxl2-3′sg-test F and R (primer set 4; sequences shown above; Supplementary Fig. 3). Founder mice were then crossed to C57BL6 mice. Genotyping of the offspring mice using PCR primers described above showed that two of the founder mice carried the template transgene ectopically in the genome in addition to the LoxP target insertions (primer set 3, 4 positive, but negative for primer set 1, 2). To genetically remove the ectopic insertions of the template transgenes, we back-crossed the founder mice C57BL6 mice for three to five generations. This process also helped wash out potential off-targets of sgRNA (off-target effects were rare in mice generated by microinjection using CRISPR/Cas9 into mouse zygotes^42^,^43^.) According to genotyping and Mendelian ratio, one male heterozygous *Loxl2*^*fl/+*^ was selected to cross with *ActinCre*^*ERT*^ and *iTcf21Cre*^*ERT*^ female mice for knockout studies. Throughout the observation period of current studies, all homozygous, heterozygous and wild-type offspring mice were grossly normal and non-distinguishable from their littermates.

### Western blot analysis

The blots were reacted with antibodies of anti-LOXL2 (1:500, ab96233, Abcam), anti-TGF-β2 (1:1,000, MAB73461, R&D Systems), anti-GAPDH (1:20,000, G9545, Sigma-Aldrich), anti-TFIIb (1:1,000, ab109106, Abcam), anti- phosphor-SMAD2 (1:1,000, 138D4, Cell Signaling), anti-SMAD2 (1:1,000, D43B4, Cell Signaling), anti-GAPDH (1:1,000, 2118, Cell Signaling), α–SMA (1:1,000, A2547, Sigma-Aldrich), total AKT (1:1,000, 4685, Cell Signaling), phospho-AKT (Ser473) (1:1,000, 4060, Cell Signaling), phospho-AKT (Thr308; 1:1,000, 4056, Cell Signaling), phospho-S6K1 (1:1,000, 9234, Cell Signaling), total S6K1(1:1,000, 9202, Cell Signaling), phospho-4EBP1 (1:1,000, 2855, Cell Signaling), PI3K*α* (1:1,000, C73FB, Cell Signaling), PI3Kβ (1:1,000, C33D4, Cell Signaling) and PI3Kδ (1:1,000, D55D5, Cell Signaling). Then followed by HRP-conjugated secondary antibodies (Jackson ImmunoResearch Laboratories, West Grove, PA, USA). Chemiluminescence was detected with ECL Western blot detection kits (GE) with LI-COR odyssey image system. Uncropped blot images are shown in Supplementary Fig. 7.

### RT–qPCR

RT–qPCR reactions were performed using SYBR green master mix (Bio-Rad, Hercules, CA, USA) with an Eppendorf realplex, and the primer sets were tested to be quantitative. Threshold cycles and melting curve measurements were performed with software. The *P* values were calculated by the Student's *t*-test. Error bars indicate standard error of mean.

### Histology and trichrome staining

Staining for LOXL2 and COL1A was performed on frozen 5 μm-thick sections of human endomyocardial biopsies. Rabbit anti-LOXL2 (1:250, Gilead Sciences) and anti-COL1A antibody (1:75, BT 21-5000-20, Biotrend Chemikalien, Germany) were used as primary antibodies, followed by secondary EnVision anti-rabbit antibody (Dako, Germany) according to manufacturer's instructions. Visualization was performed with carbazol-staining solution and background staining was performed with a Mayer's hemalum solution (Sigma-Aldrich, Germany). Digital image analysis was conducted on a Leica DMRB microscope (Leica Microsystems, Germany) at a × 200 magnification.

As described previously^44^, adult mice hearts were fixed overnight in 4% paraformaldehyde in phosphate-buffered saline (PBS). Subsequently, they were dehydrated through an ethanol series, treated with xylenes and embedded in paraffin wax overnight with several changes. The hearts were oriented for transverse sections and cut in 7 μm sections using a Leica microtome. Trichrome staining was performed following the protocol of trichrome stain (Masson) kit (HT15) from Sigma-Aldrich. Deparaffinize the slides to deionized water, then mordant in preheated Bouin's Solution (HT10-1) at 56 °C for 15 min. Cool the slides in tap water (18–26 °C) and wash in running tap water to remove yellow colour from sections. Put the slides in Working Weigert's Iron Haematoxylin Solution for 5 min, Biebrich Scarlet-Acid Fucshin (HT15–1) 5 min, working Phosphotungstic/Phosphomolybdic Acid Solution for 5 min, Aniline Blue Solution (HT15–4) for 5 min, 1% acetic acid for 2 min. Finally, rinse slides, dehydrate through alcohol, clear in xylene and mount. The imaging was performed using Leica microscope. The following primary antibodies were used for mice tissue immunostaining: anti-LOXL2 (1:100, Rabbit Polyclonal antibody, Gilead Sciences), anti-CD4 (1:100, sc-13573, Santa Cruz), anti-COL1A (Chemicon, MA, USA) and α–SMA (1:200, A2547, Sigma-Aldrich). Immunohistochemistry was conducted using biotinylated secondary antibodies (anti-rabbit IgG, 1:250; anti-mice IgG, 1:250; Jackson Immunoresearch), VECTASTAIN Elite ABC Kit (PK-6200, Vector Laboratories) and DAB developing reagents (DAKO). Imaging was performed using a Leica microscope.

### Collagen content measurements

To determine total cardiac collagen content, we measured the content of hydroxyproline, a major component of collagen, as described^39^. Left ventricles were hydrolysed in hydrochloric acid HCl, and hydroxyproline level was measured by a colorimetric method using assay kits from Qickzyme. The total collagen content was calculated from the hydroxyproline content of collagen standards. To determine soluble collagen content, left ventricles were solubilized in pepsin-acid solution, and the supernatants were precipitated by trichloroacetic acid solution. Dried pellets were then hydrolysed in HCl, and hydroxyproline measured. The soluble collagen content was calculated from the hydroxyproline content of collagen standards. The insoluble collagen content was determined by subtracting the amount of soluble collagen from total collagen.

### LOXL2 siRNA knockdown in human primary cardiac fibroblasts

Human primary cardiac fibroblasts were isolated from the ventricles (Lonza, Walkersville Inc., Walkersville, MD, USA; CC-2904). These cells were cultured in FGM-2 BulletKits media (Lonza, CC-4526) at 37 °C and 5% CO_2_. Human LOXL2 and control siRNA (Qiagen, SI00036134 and SI03650318) were transfected into human fibroblasts with lipfectmain RNAiMAX. After 48 h, the cells were collected, culture media collected, cells lysed and total RNA extracted for further analyses. The levels of mRNA were measured by RT–qPCR using primers for individual genes or TGF-β/BMP signalling arrays (Qiagen). The siRNA for LOXL2 was 5′-CCGGAGTTGCCTGCTCAGAAA-3′. Primers for human LOXL2 (QT00019425), TGF-β1 (QT00000728), TGF-β2 (QT00025718) and TGF-β3 (QT00001302) were from Qiagen. The levels of TGF-β in the culture media were measured with Milliplex Map TGF-β 3-Plex (EMD Millipore).

### LOXL2 overexpression in human primary cardiac fibroblasts

Adenoviral vectors expressing human LOXL2 or GFP were purchased from Applied Biological Materials. Human cardiac fibroblasts were transduced with adenovirus for 6 h, and the cells were cultured in FGM-2 medium with 2% FBS for 48 h and culture medium, cell lysates and total RNA were isolated for measuring mRNA and TGF-β levels.

### Gap closure assay

A gap closure assay was used to study the migration of human ventricular fibroblasts (Lonza Walkersville Inc., Walkersville, MD, USA). The cells were seeded into collagen I-coated 24-well plates (Life Technologies Corporation, Grand Island, NY, USA) at the density of 10^5^ cm^−2^ cells. The culture was grown to high confluency, and then the cells were serum-starved for 24 h before the treatment. The cell monolayer was scratched with a sterile pipette tip to remove cells and create a gap. The average width of the scratch was 876±38 μm. The culture medium was replaced with fresh media immediately after scratching. The anti-LOXL2 antibody (AB0023) was added at a concentration of 10 μg ml^−1^, and the cell migration was activated with TGF-β2 (5 ng ml^−1^). A mouse IgG1 antibody was used as an isotype control. The fibroblasts were allowed to migrate into the gap for approximately 16 h. After that, the cells were loaded with fluorescent dye calcein AM (0.2 μM, Life Technologies Corporation) at 37 °C for 30 min. Once the dye loading was completed, fluorescence images were acquired using Zeiss LSM 5 Pascal confocal microscope equipped with × 4 objective. Calcein fluorescence was excited with an Argon laser at 488 nm and detected at wavelengths >505 nm using a long pass filter. To count the migrated cells, the images were analysed using Image J (NIH) software.

### Statistical analysis

Comparisons were performed with the Mann–Whitney *U*-test used for non-parametric continuous variables or the Student's *t*-test for parametric variables and Fisher's exact test for categorical variables. Correlation of parameters was performed with Pearson or Spearman coefficients, and regression analysis was used for exact relations. Statistical significances were calculated using GraphPad Prism 5 and 7 (GraphPad Software, La Jolla, CA, USA). A value of *P*<0.05 was considered statistically significant.

### Data availability

The authors declare that the data supporting the findings of this study are available within the article and its Supplementary Information files and from the corresponding author on request.

## Additional information

**How to cite this article:** Yang, J. *et al*. Targeting LOXL2 for cardiac interstitial fibrosis and heart failure treatment. *Nat. Commun.* **7,** 13710 doi: 10.1038/ncomms13710 (2016).

**Publisher's note**: Springer Nature remains neutral with regard to jurisdictional claims in published maps and institutional affiliations.

## Supplementary Material

Supplementary Information Supplementary Figures 1-7 and Supplementary Table 1.

## Figures and Tables

**Table 1:** Demographics and diagnosis of control subjects and patients.

**Age**	**Gender**	**Ethnicity**	**Diagnosis**
*a Control subjects*			
49†	M	White	NF*
54†	M	White	NF
52†	M	White	NF
68	M	White	NF
52	M	White	NF
64	M	White	NF
41	M	White	NF
41	M	White	NF
			
*b Patients with cardiomyopathy*			
49†	M	White	ISCH
54†	M	White	ISCH
58†	M	White	ISCH
56	M	White	ISCH
61	M	White	ISCH
65	M	White	ISCH
62	F	White	ISCH
57	F	White	ISCH
62	M	White	ISCH
34†	M	White	IDCM
41†	M	White	IDCM
47†	M	White	IDCM
61	M	White	IDCM
55	M	White	IDCM
62	M	White	IDCM
47	M	White	IDCM
66	M	White	IDCM
65	M	White	IDCM
53	M	White	IDCM
63	M	White	IDCM
55	M	White	IDCM
46	M	White	IDCM

(**a**) Demographics and diagnosis of control subjects of heart organ donors and (**b**) patients with ischaemic or idiopathic dilated cardiomyopathy. IDCM, idiopathic dilated cardiomyopathy; ISCH, ischaemic cardiomyopathy.

^*^NF: non-failure, normal heart function.

^†^Sample for tissue immunostaining.

**Table 2:** Demographics, echocardiographic and hemodynamic parameters in control subjects and patients diagnosed with HFpEF.

	**Control (*****n*****=15)**	**HFpEF (*****n*****=24)**	***P*** **value**
*Demographics*			
Age	47.3±6.3	52.5±7.4	0.07
NT-proBNP (pg ml^−1^)	61.44±54.26	198.6±191	0.047
Weight (kg)	81.9±21	78.1±15.7	0.833
6-min walk test (m)	540.8±47	339.59±58.69	<0.001
NYHA II/III	0/0	17/7	—
Women/men	4/11	13/11	0.11
Hypertension	1	19	<0.001
Diabetes mellitus	0	4	0.146
Obesity	2	8	0.263
Dyslipidaemia	4	13	0.116
Smoker	4	6	1.0
			
*Echocardiographic measurements*			
LA parasternal (mm)	35.6±4.9	37.5±6.5	0.319
LVEDD (mm)	51.3±2.63	49.5±6.74	0.491
IVSd (mm)	10.8±1.66	12±3.7	0.472
LVPWd (mm)	10.1±1.34	11.34±3.8	0.35
LVEF (%)	59±12	58.3±8.6	1.0
			
*Mitral inflow*			
E (m s^−1^)	0.81±0.19	0.83±0.2	0.831
A (m s^−1^)	0.68±0.42	0.82±0.18	0.038
E/A	1.25±0.42	1.1±0.37	0.138
DT (ms)	197.4±59.9	215.7±47.9	0.261
IVRT (ms)	103.6±28.9	96.2±17.7	0.465
			
*Pulmonary vein flow*			
Systolic, S (m s^−1^)	0.53±0.07	0.62±0.1	0.432
Diastolic, D (m s^−1^)	0.66±0.12	0.56±0.13	0.4
Ar (m s^−1^)	0.32±0.32	0.35±0.5	0.629
S/D ratio	0.83±1.9	1.1±0.27	0.393
			
*Tissue velocity imaging*			
S′ mean (cm s^−1^)	9.13±3.6	7.7±2.4	0.73
E′ mean (cm s^−1^)	10.9±3.9	6.5±2.1	<0.001
A′ mean (cm s^−1^)	8.4±2.4	7.5±2.9	0.262
E′/A′ mean	1.33±0.5	1±0.38	0.061
E/E′ mean	8.4±2.17	14.9±8.04	<0.001
			
*Hemodynamic measurements*			
LVEDP (mm Hg)	11.1±3.8	15.4±5.8	0.031

AR: atrial reversal; DT, deceleration time; IVRT, isovolumic relaxation time; IVSd, interventricular septum thickness (measured in diastole); LA, left atrium; LVEDP, left ventricular end-diastolic pressure; LVEF, left ventricular ejection fraction; LVEDD, left ventricular end-diastolic diameter; LVPWd, left ventricular posterior wall thickness (measured in diastole); NT-proBNP, N-terminal pro-brain natriuretic peptide; NYHA, New York Heart Association.
