# Supplementary material for: Targeting LOXL2 for cardiac interstitial fibrosis and heart failure treatment
Source: Nat Commun. 2016 Dec 14;7:13710. doi: 10.1038/ncomms13710 (PMC5171850; doi:10.1038/ncomms13710)
Supplement: Supplementary Information — Supplementary Figures 1-7 and Supplementary Table 1. [file ncomms13710-s1.pdf]

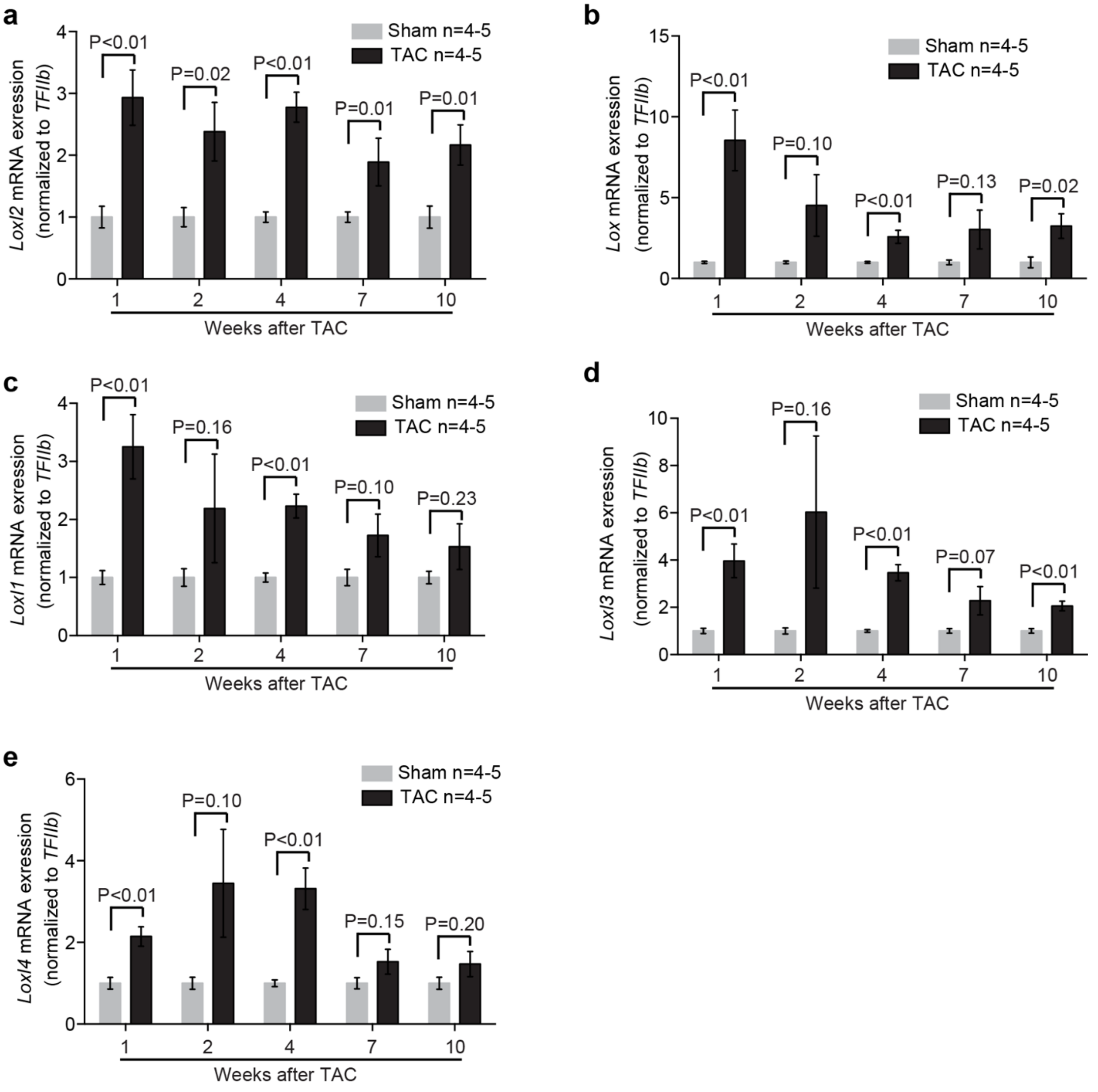

Supplementary Figure 1. *Lox*, *Loxl1–4* mRNA detection in mouse heart tissues after TAC. (**a-e**) Quantitation of *Loxl2* (**a**), *Lox* (**b**), Lox1 (**c**), *Lox3* (**d**), and *Lox4* (**e**) mRNA in mouse heart ventricles 1–10 weeks after sham/TAC operation, normalized to *TFIIb*. *n*=4–5 mice per group. *P*-value: Student's t-test. Error bar: SEM.

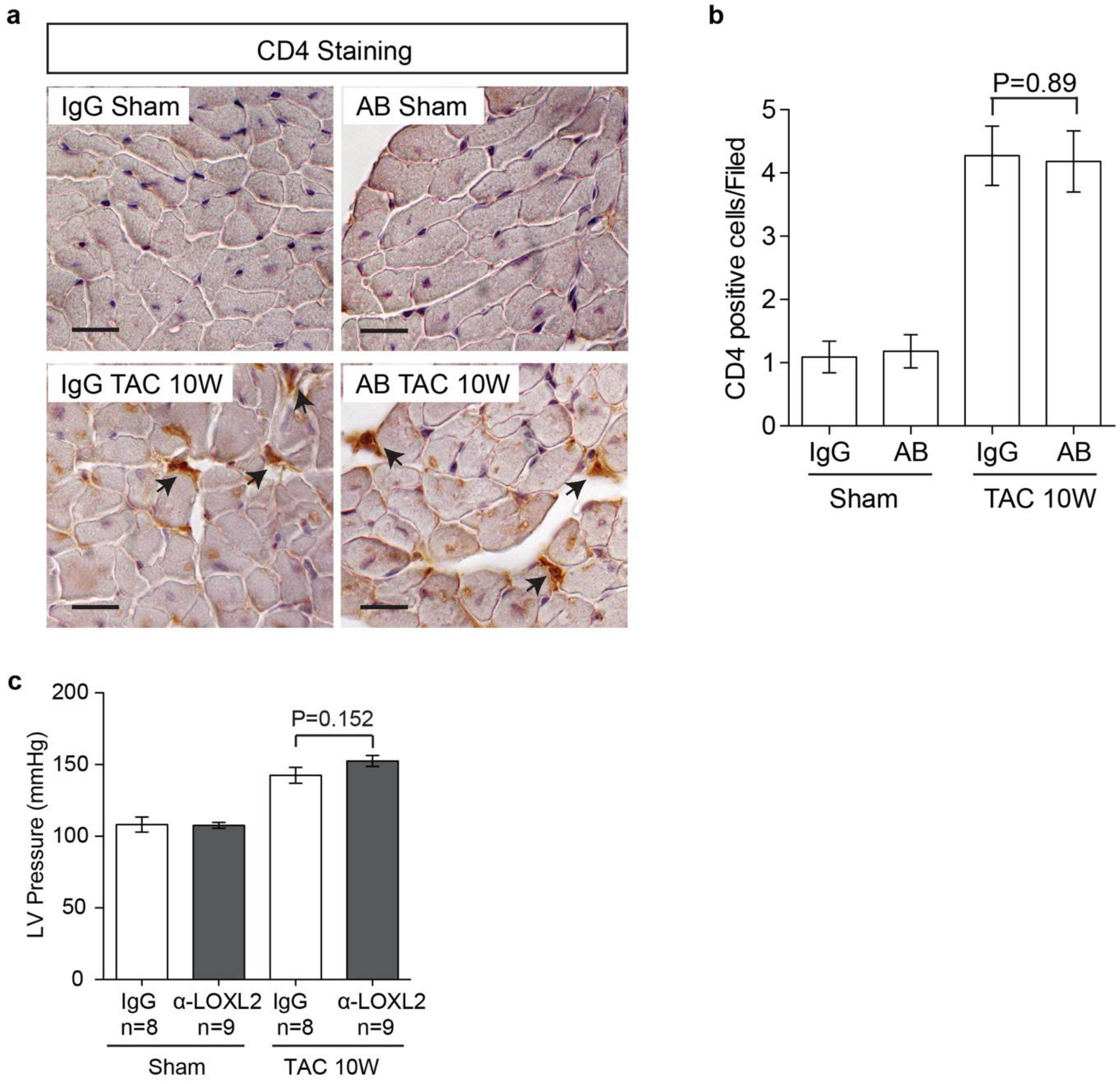

Supplementary Figure 2. LOXL2 antibody treatment has no effects on immune cell infiltration or hypertrophy. (**a**) Immunostaining of CD4 in the heart 10 weeks after sham/TAC operation of IgG or LoxL2 antibody treated mice. Scale bars, 10μm. Blue: Hematoxylin. Brown: CD4. (**b**) Quantitation of CD4 positive cells/field. *n*=4 mice per group. *P*-value: Student's t-test. Error bar: SEM. (**c**) Quantitation of left ventricular pressure of IgG1- or α-LOXL2-treated mice 10 weeks after sham or TAC operation. *n*=8-9 per group. *P*-value: Student’s t-test. Error bar: SEM.

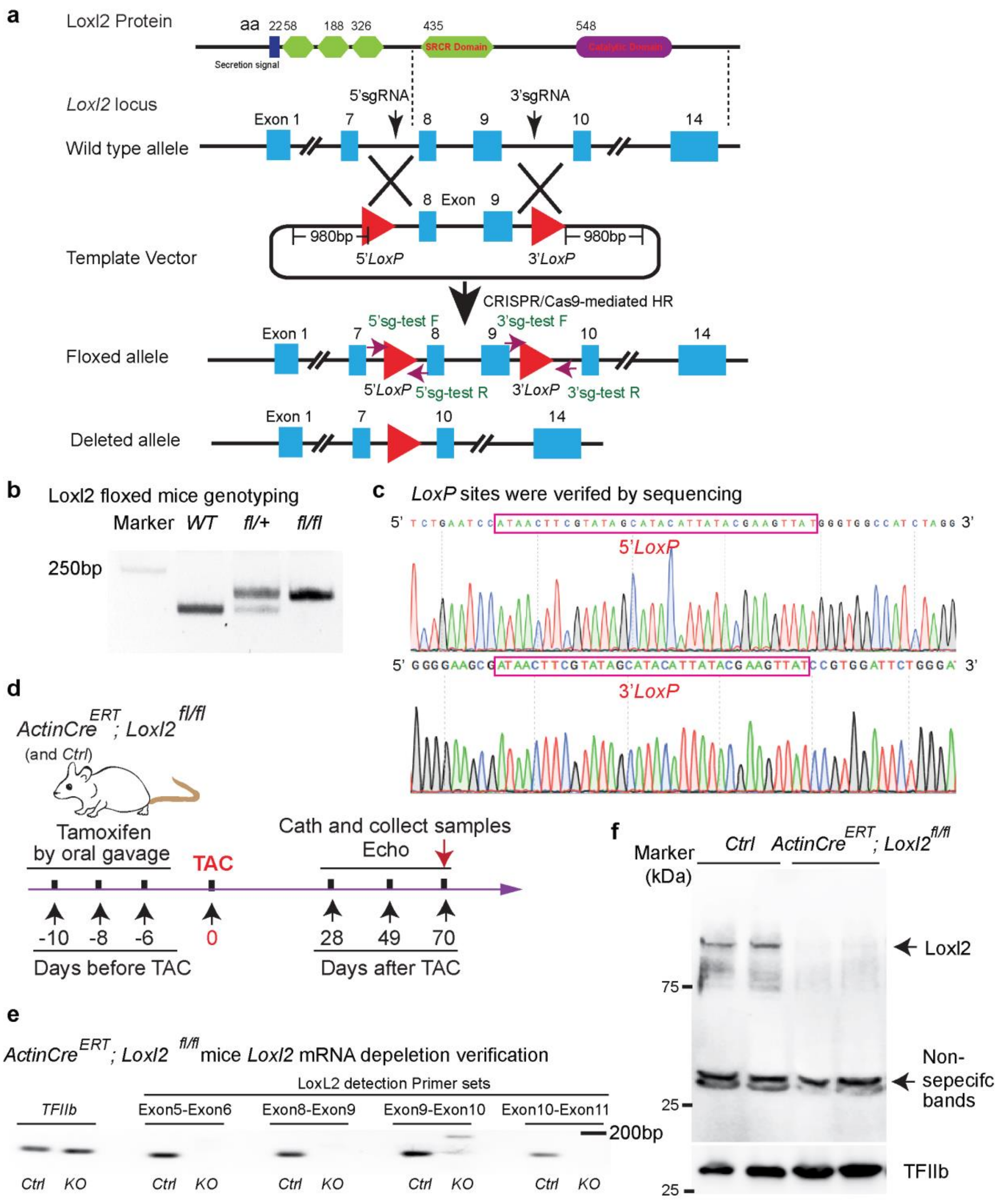

Supplementary Figure 3. Generation of *Loxl2* floxed alleles by CRISPR/Cas9 editing. (**a**) Exon 8 and 9 are flanked by 5' and 3'LoxP sequences in one homologous recombination (HR) reaction to generate LoxP-flanked (floxed) Loxl2 allele. Once

recombined by the Cre recombinase, the *LoxP* sequences loop out and cause the deletion of exon 8 and 9 from the genome (deleted allele), resulting in disruption of gene regions encoding the SRCR and the downstream catalytic domains. (**b, c**) Identification of mouse genotypes by PCR (**b**) with the indicated primers (F1, R1) and sequencing verification of 5' and 3' *LoxP* sites (**c**). WT, wild-type mice; fl/+, *Loxl2* $^{fl/+}$ mice; fl/fl, *Loxl2*$^{fl/fl}$ mice. (**d**) Experimental scheme for *Loxl2* deletion by Tamoxifen administration. (**e, f**) qPCR and western blotting of heart tissues of *ActinCre*^{ERT}*;Loxl2* $^{fl/fl}$ KO mice, using PCR primers that target non-deleted regions of Loxl2 (**e**) and polyclonal antibody against Loxl2 (**f**).

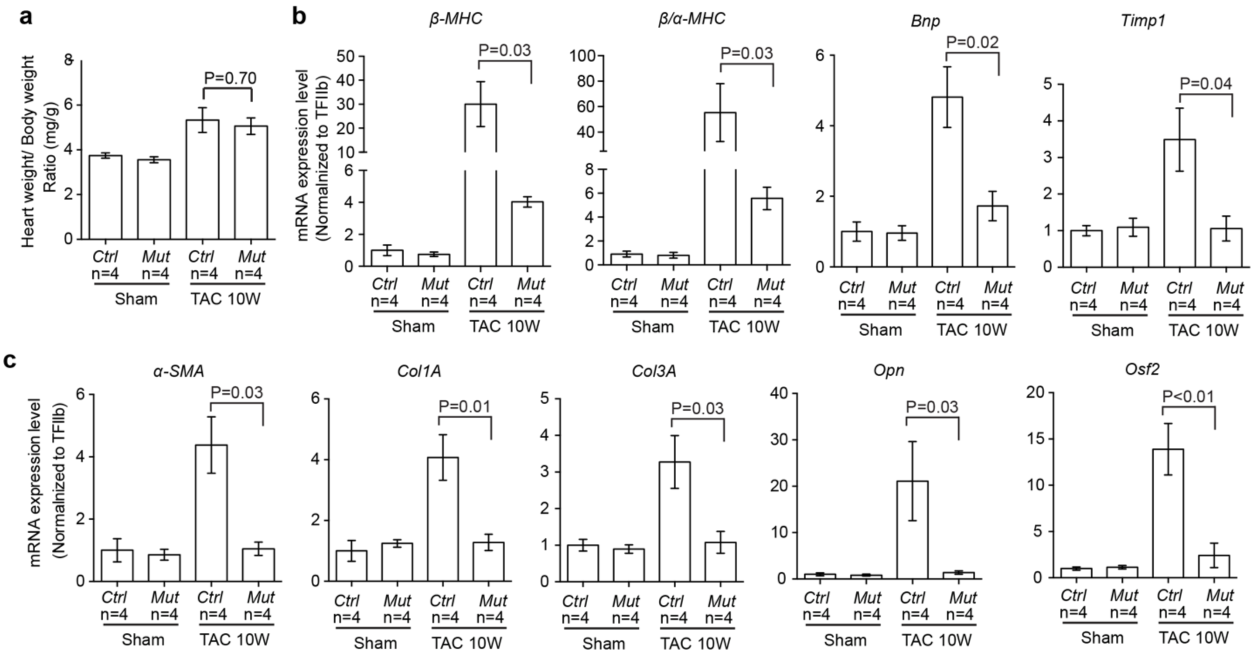

Supplementary Figure 4. Loxl2 global knockout prevents TAC-induced cardiac interstitial fibrosis and heart dysfunction. (**a**) Ventricle–body weight ratio of hearts harvested 10 weeks after sham or TAC operation. *Ctrl*: *ActinCre*^{ERT}*;Loxl2* $^{fl/+}$, *Loxl2* $^{fl/fl}$ or *Loxl2* $^{fl/+}$. *Mut*: *ActinCre*^{ERT}*;Loxl2* $^{fl/fl}$. *n*=4 per group. *P*-value: Student's t-test. Error bar: SEM. (**b**) Quantitation of cardiac stress marker genes-*β-MHC*, *β/α-MHC*, *Bnp* and *Timp1* in the control and mutant mice hearts 10 weeks after sham or TAC operation. *Ctrl*: *ActinCre*^{ERT}*;Loxl2* $^{fl/+}$, *Loxl2* $^{fl/fl}$ or *Loxl2* $^{fl/+}$. *Mut*: *ActinCre*^{ERT}*;Loxl2* $^{fl/fl}$. *n*=4 per group *P*-value: Student's t-test. Error bar: SEM. (**c**) Quantitation of cardiac fibrosis marker genes-*α-SMA*, *Col1A*, *Col3A*, *OPN*, and *OSF2* in the control and mutant mice hearts 10 weeks after sham or TAC operation. *Ctrl*: *ActinCre*^{ERT}*; Loxl2* $^{fl/+}$, *Loxl2*$^{fl/fl}$ or *Loxl2* $^{fl/+}$. *Mut*: *ActinCre*^{ERT}*; Loxl2*$^{fl/fl}$. *n*=4 per group. *P*-value: Student's t-test. Error bar: SEM.

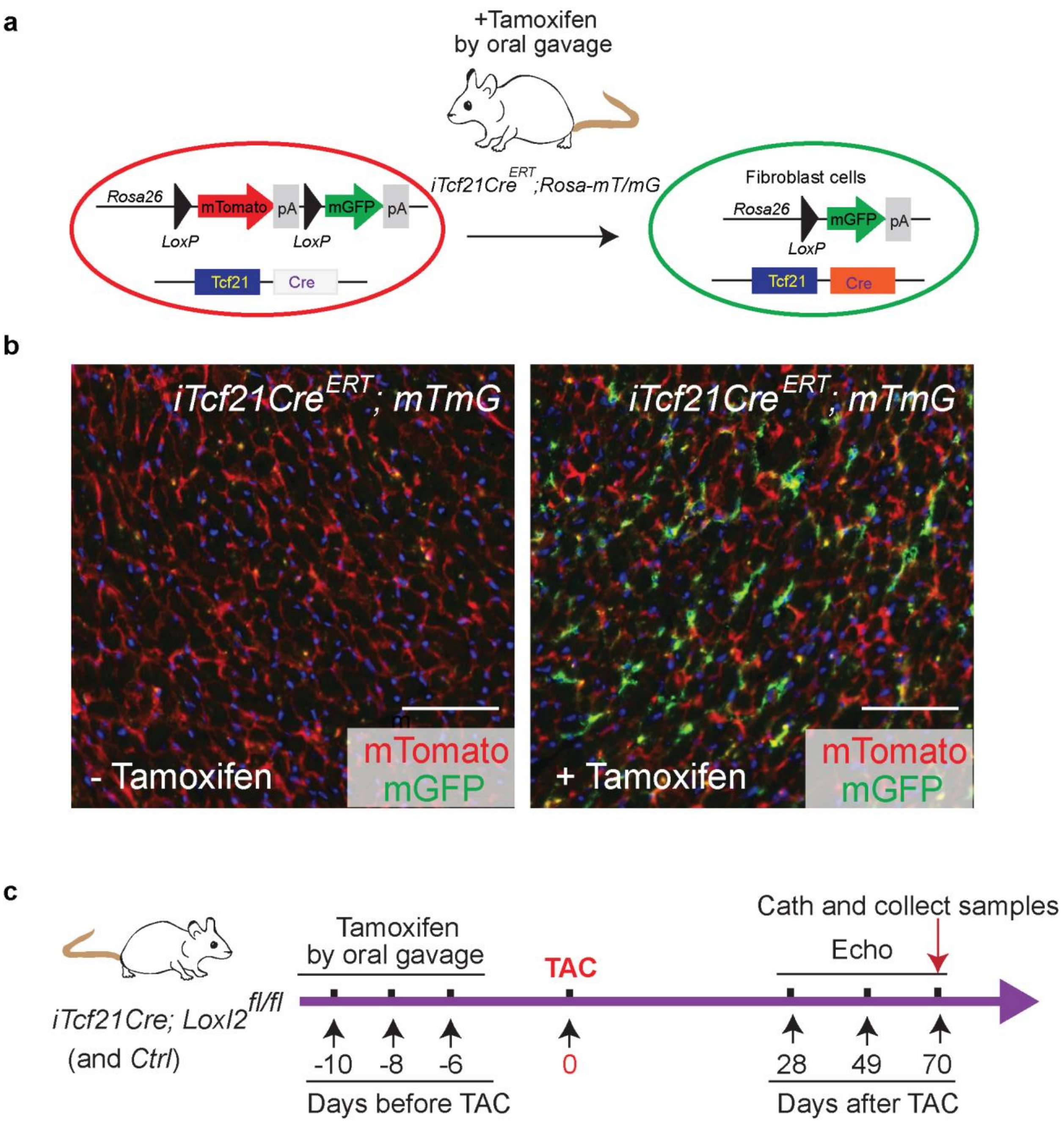

Supplementary Figure 5. Tissue-specific knockout of Loxl2 in cardiac fibroblasts. (**a**) Genetic scheme for tissue-specific knockout of Loxl2 in cardiac fibroblasts. (**b**) Fluorescent images of *iTcf21Cre*^{ERT};*mTmG* mice with or without tamoxifen treatment. Green: GFP indicating Cre activity in fibroblasts in cardiac interstitium. Scale bars, 20μm. Red: Tomato indicating cellular sites that do not have Cre activity. (**c**) Experimental scheme of *Cre* induction in cardiac fibroblast by Tamoxifen.

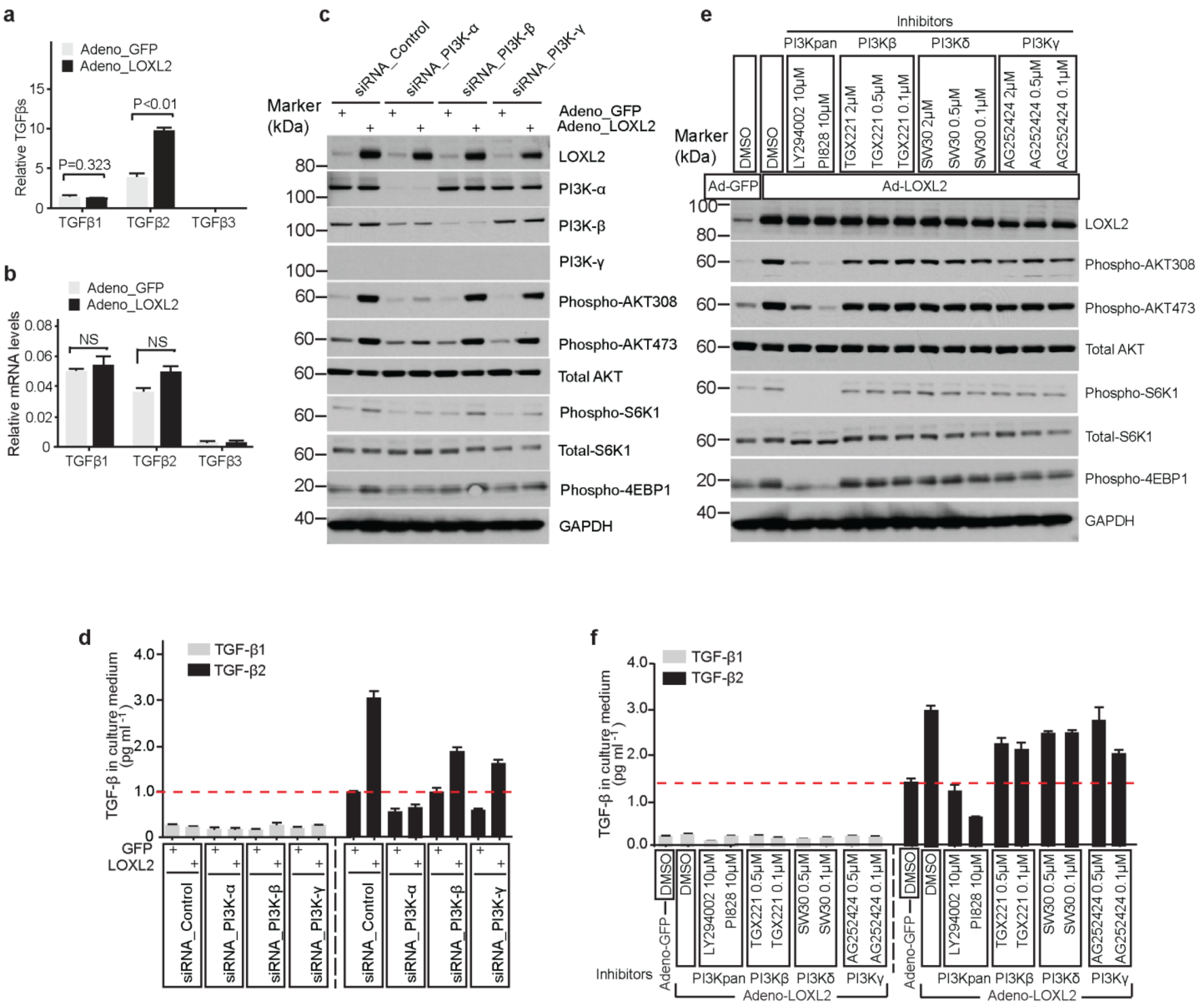

Supplementary Figure 6. LOXL2 promotes TGF-β2 production through PI3Kα/AKT/mTORC1. (**a, b**) TGF-β production (**a**) and quantitation of *TGF-β* mRNAs (**b**) in human cardiac fibroblasts infected with adenovirus carrying LOXL2 or GFP. *n*=4, *P*-value: Student's t-test. Error bar: SEM. (**c**) Western blot of LOXL2, PI3Kα, PI3Kβ, PI3Kδ, p-AKT, AKT, p-S6K1, S6K1, and p-4E-BP1 with or without PI3Kα, PI3Kβ, and PI3Kδ siRNA in human cardiac fibroblasts infected with GFP/LOXL2. (**d**) TGF-β production in human cardiac fibroblasts with or without PI3Kα, β, and δ siRNA. *n*=4, *P*-value: Student's t-test. Error bar: SEM. (**e**) Western blot of LOXL2, p-AKT, AKT, p-S6K1, S6K1, and p-4E-BP1 with or without PI3K, PI3Kβ and δ inhibitors in cells infected with GFP/LOXL2. (**f**) TGF-β production in human cardiac fibroblasts with or without PI3K, PI3Kβ, and δ inhibitors. *n*=4, *P*-value: Student's t-test. Error bar: SEM.

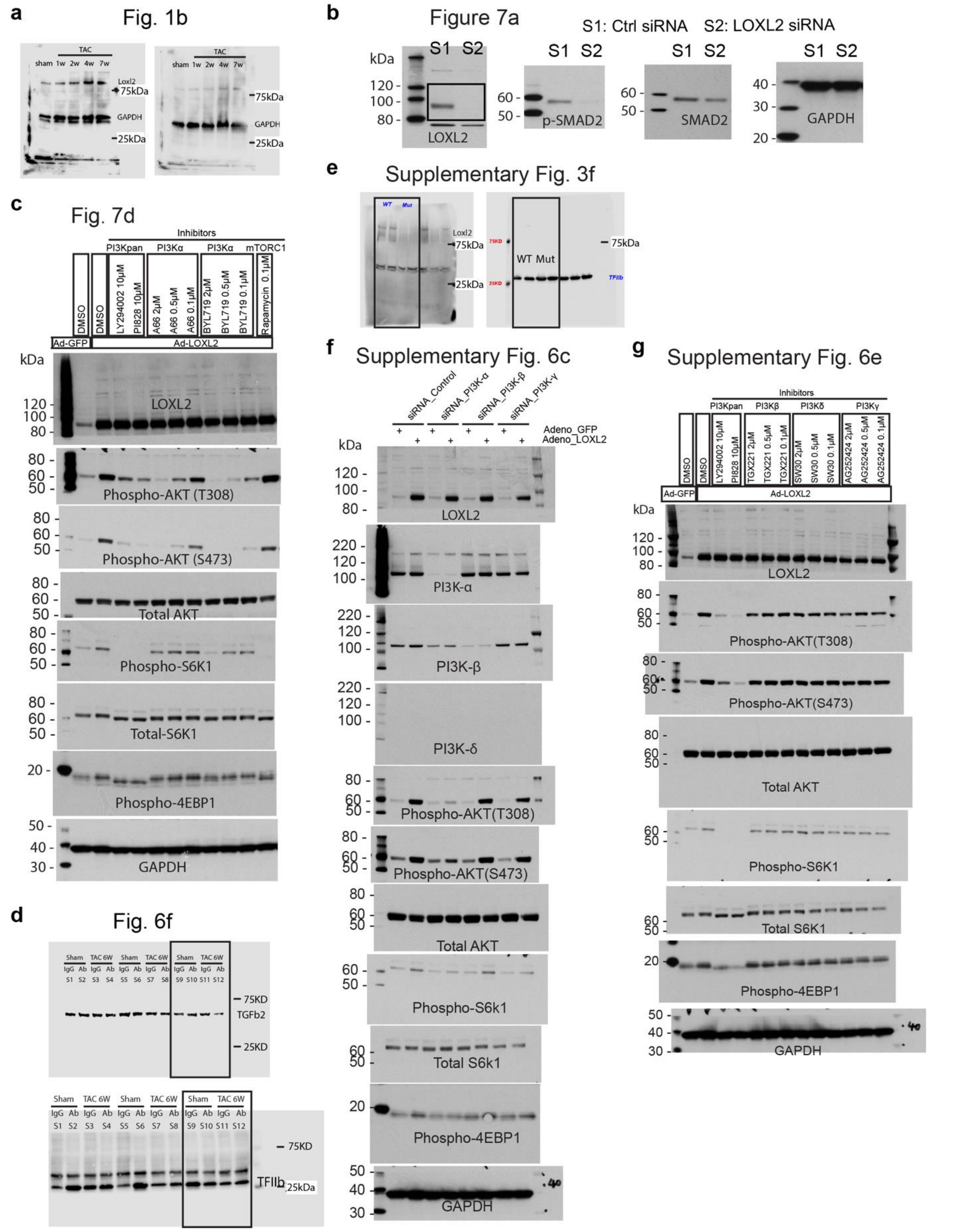

Supplementary Figure 7. The uncropped images of Western Blot Analyses. (**a-c**) The

original western blot images of Fig. 1b (**a**), Fig. 7a (**b**), Fig. 7d (**c**) and Fig. 7f (**d**). (**e**-**g**) The original western blot images of supplementary Fig. 3f (**e**), supplementary Fig. 6c (**f**), and supplementary Fig. 6e (**g**).

Supplementary Table 1. Demographics of control subjects and patients with HFrEF and HFpEF for biomarker studies.

Control Subjects

Age	Gender	Ethnicity	CAD*
66	M	White	Yes
60	M	White	Yes
59	M	White	Yes
62	F	White	No
58	M	White	No
40	F	White	No
53	F	White	No
42	F	White	No
68	F	White	No
53	M	White	No
53	F	White	No
57	F	White	Yes
38	F	White	No
57	F	White	No
71	M	White	No
56	M	White	No
48	F	White	No
70	M	White	Yes
88	F	White	No
65	M	White	No
79	M	White	No
49	M	Black	No
64	M	White	No
77	F	White	No
62	F	White	No
64	M	White	No
70	M	White	No
53	F	White	No
74	F	White	No
69	M	White	No
71	F	White	No
81	F	White	No
60	F	White	No
82	F	White	No

*CAD: Coronary Artery Disease

Patients with HFrEF

Age	Gender	Ethnicity	CAD
56	F	Black	No
71	M	White	Yes
69	M	White	Yes
76	F	White	Yes
59	M	Black	No
56	F	Black	Yes
68	F	White	Yes
58	M	Black	No
58	F	White	Yes
65	F	White	Yes
54	M	White	Yes
76	M	White	Yes
59	M	White	Yes
44	F	White	Yes
73	M	White	Yes
89	F	White	No
67	M	White	No
80	M	White	No
45	M	Black	No
59	M	White	Yes
79	F	White	Yes

Patients with LVAD (EF≤35%) HFrEF**

Age	Gender	Ethnicity	CAD
53	M	White	Yes
35	M	White	No
40	M	White	Yes
55	M	White	Yes
60	M	White	Yes
73	M	White	No
68	F	White	No
57	M	White	No
30	M	White	Yes
34	M	White	No

**LVAD: Left Ventricular Assistant Device

Patients with LVAD (EF≥40%)

Age	Gender	Ethnicity	CAD
35	F	White	No
53	F	White	Yes
26	F	White	No
52	F	White	No
43	M	White	No

Patients with HFpEF

Age	Gender	Ethnicity	CAD
73	F	White	Yes
38	M	White	
41	M	White	
68	F	White	Yes
72	F	White	Yes
82	M	White	Yes
78	M	White	Yes
79	F	Black	Yes
61	M	White	Yes
93	F	White	No
60	M	Black	No
69	M	White	Yes
24	M	White	No
35	M	White	No
82	F	Black	No
62	F	White	No
62	M	White	Yes
73	M	White	Yes
56	F	White	Yes
72	F	White	Yes
70	M	White	Yes
76	F	White	Yes
90	F	White	Yes
57	F	White	Yes
85	F	White	No
